# Recovery of Soils From Acidic Deposition May Exacerbate Nitrogen Export From Forested Watersheds

**DOI:** 10.1029/2019jg005036

**Published:** 2019-10-24

**Authors:** Gregory B. Lawrence, Sara E. Scanga, Robert D. Sabo

**Affiliations:** 1 U.S. Geological Survey, New York Water Science Center, Troy, NY, USA; 2 Department of Biology, Utica College, Utica, NY, USA; 3 Oak Ridge Institute for Science and Education, Washington, DC, USA; 4 U.S. Environmental Protection Agency, Office of Research and Development, Washington, DC, USA

## Abstract

Effects of ambient decreases in N deposition on forest N cycling remain unclear as soils recover from acidic deposition. To investigate, repeated soil sampling data were related to deposition, vegetation, and stream data, for 2000–2015 in North and South Buck Creek watersheds, in the Adirondack region of New York, USA. In 63 other Adirondack streams, ${{\text{NO}}_{3}}^{-}$ concentrations were also compared between 2004–2005 and 2014–2015, and a link between soil calcium and stream ${{\text{NO}}_{3}}^{-}$ was investigated using data from 387 Adirondack streams that were sampled in either 2003–2005 or 2010–2011. No trends in N export or ${{\text{NO}}_{3}}^{-}$ concentrations were observed in either Buck watershed despite a 45% decrease in N deposition, although South Buck N export was 2 to 3 times higher than in North Buck, where 48% of deposited N was accounted for by accumulation in the upper soil. In marked contrast, the upper profile in South Buck showed a net loss of N. Increased decomposition appeared likely in South Buck as those soils are adjusted to lower levels of acidifying S deposition, whereas decomposition increases in North Buck were likely suppressed by high levels of natural organic acidity. Stream ${{\text{NO}}_{3}}^{-}$ concentrations in Buck watersheds bracketed regional results and were consistent with the regional streams that showed no overall change in ${{\text{NO}}_{3}}^{-}$ concentrations between 2004 and 2014. A negative correlation observed between ${{\text{NO}}_{3}}^{-}$ concentration and watershed buffering capacity expressed as the ratio of ${\text{Ca}}^{2+}$ to ${{\text{SO}}_{4}}^{2-}$ also suggested that stream ${{\text{NO}}_{3}}^{-}$ concentrations were elevated where soil Ca depletion had occurred.

## 1. Introduction

Deposition of atmospheric nitrogen (N) on forested watersheds continues to receive a high level of research interest due to the various roles that N can play in major environmental issues such as forest health and productivity and carbon (C) sequestration in soil. Atmospheric N deposition (hereafter N deposition) may increase C fixation where N is growth limiting (Sutton et al., 2008), but where mineral nutrient availability is low, N deposition can result in an excess of available N (N saturation) that can decrease growth and increase mortality of trees (Fenn et al., 1998), as well as acidify surface waters (Driscoll et al., 2001). The relationship of N deposition to stream water N has been shown to be related to measures of ecosystem N status, such as forest floor C:N ratios, which can be a function of a variety of factors (Johnson & Turner, 2014). Nevertheless, in experimentally controlled forest catchments that were releasing high amounts of N to soil waters, artificial removal of N from atmospheric deposition resulted in rapid decreases in N loss from the catchments (Tietema et al., 1998), and efforts continue in North America and Europe to reduce N emissions for the express purpose of reducing surface water N concentrations (Greaver et al., 2012).

In landscapes with low acid-buffering capacity, which occur throughout much of eastern North America and northern Europe, N often plays a larger role as an acidifier than as a nutrient (Driscoll et al., 2001; Gundersen, 1985). In many of these landscapes, high levels of acidic deposition from sulfur (S) and nitrogen (N) over the past decades substantially lowered the availability of calcium (Ca) and other mineral nutrients through acid leaching driven by H_2_SO_4_ and HNO_3_ (Johnson et al., 2008; Lawrence et al., 2005). Depletion of Ca not only lowered the already low acid-buffering capacity of these soils but also increased the possibility that Ca would become growth limiting for tree species with high Ca demand (Engel et al., 2016; Long et al., 2009), such as sugar maple (*Acer saccharum* Marsh.) and red spruce (*Picea rubens* Sarg.), thereby increasing the susceptibility of forest ecosystems to N saturation. Because N in excess of ecosystem demand can readily produce HNO_3_ through nitrification, depletion of Ca is exacerbated by the acid leaching that accompanies surplus N (Fenn et al., 1998), further worsening the nutrient imbalance. Depletion of Ca by acidic deposition is also problematic because it leads to mobilization of aluminum (Al) within the rooting zone in forms that are detrimental to trees, lowering growth (Halman et al., 2013; Shortle & Smith, 1988) and therefore N demand. Despite these potential links between Ca and N availability, Ca-N interactions remain poorly understood. For example, experimental addition of Ca in the form of wollastonite (CaSiO_3_) to a Ca-depleted watershed in New Hampshire, USA, resulted in increased forest growth (Battles et al., 2014) and decreases in microbial and inorganic N pools (Groffman et al., 2006) but also eventual increases in watershed export of N (Rosi-Marshall et al., 2016).

High rates of N loss and associated stream acidification in forested watersheds impacted by acidic deposition have been well documented (Driscoll et al., 2001). However, accumulating evidence suggests that decreased pH and increased Al mobilization led to increased retention of soil organic matter, which includes N and thereby may have offset some of the N loss associated with N saturation. For example, acid addition (as H_2_SO_4_) to podzols (Spodosols in U.S. taxonomy) with histories of either low or high acidic deposition levels decreased mobilization of dissolved organic C (DOC), whereas base addition increased DOC mobilization (Evans et al., 2012). Also, increasing DOC trends in surface waters in the northeastern United States, eastern Canada, Scandinavia, and the United Kingdom were ascribed to increased solubility of soil organic matter from increasing pH caused by decreasing acidic deposition—a reversal of the prior pH depression that decreased organic C solubility (Monteith et al., 2007). These findings are supported by laboratory and modeling results that demonstrated reduced organic C solubility with decreased pH (Clark et al., 2006) and increased organic Al complexation (De Wit et al., 1999). Furthermore, increased organic matter stabilization by organic Al complexation was observed in decomposition studies conducted in acidic forest soils (Kunito et al., 2016; Scheel et al., 2007). The past increases in forest floor Al that resulted from acidic deposition (Johnson et al., 2008; Lawrence et al., 1995; Warby et al., 2009) also point to increased soil C sequestration.

Accumulation of organic C in forest soils from acidic deposition suggests that with decreases in acidic deposition, this trend might reverse and decreases in forest floor C in recovering soils have been documented in Europe (Oulehle et al., 2011) and the United States (Lawrence et al., 2012). Loss of organic C from the forest floor has the potential to affect N dynamics in a number of ways by releasing organic-bound N. Despite the considerable attention given to the effects of N deposition on N cycling in forest ecosystems, the variety and complexity of observed responses have left an incomplete understanding of how forested watersheds are responding to decreasing N deposition. Further uncertainties have developed with the beginnings of soil recovery from decreased S deposition (Lawrence et al., 2015). Much of the research on N deposition effects has utilized manipulation experiments that have defined the range of potential responses based on increases and decreases of controlled N inputs. However, much less information is available on the long-term effects of ambient changes in N deposition, particularly within the context of emerging soil recovery linked to decreased S deposition.

An opportunity to evaluate the effects of decreasing N deposition on forest N dynamics in watersheds where soils are undergoing recovery from acidic deposition was provided by monitoring streams, soils, and trees in two watersheds and regional stream surveys conducted in 2004–2005, 2010–2011, and 2014–2015 in the Adirondack region of New York, USA. The Adirondack region has experienced pronounced changes in both terrestrial and aquatic ecosystems from some of the highest levels of S and N deposition in the United States (Driscoll et al., 2001; Lawrence et al., 2018) and has also begun to show indications of recovery in soils (Lawrence et al., 2015) and streams (Lawrence, Burns, & Riva-Murray, 2016) from declining S deposition. In a previous study, export of N (largely in the form of ${{\text{NO}}_{3}}^{-}$) from these same two watersheds unexpectedly did not exhibit trends from 2000 to 2011 (*P* > 0.10) despite a marked decrease in wet N deposition (Sabo et al., 2016). However, the cause of the continued watershed export of N was unclear, as was the relationship of this response to other forested watersheds that have been impacted by S and N deposition.

To advance our understanding of how ecosystem N dynamics are responding to declining N deposition as recovery from acidic deposition proceeds, the study presented herein was designed with the following objectives: (1) evaluate potential controls on watershed N export from 1999 to 2015 in two neighboring watersheds (North and South Buck Creek) with contrasting soils and vegetation, (2) determine if stream ${{\text{NO}}_{3}}^{-}$ concentrations changed in approximately 60 streams sampled in 2004 and 2014 in the western Adirondack region, and (3) relate findings from the comparative watershed analysis to the spatial variability of ${{\text{NO}}_{3}}^{-}$ concentrations in headwater streams throughout the 24,000-km^2^ Adirondack region.

## 2. Methods

### 2.1. Study Region

The study was conducted within the Adirondack ecological region (McNab & Avers, 1994), which roughly corresponds to the boundary of the Adirondack State Park (Figure S1 in the supporting information). The study region is characterized by rugged terrain formed by repeated glaciations that last receded approximately 10,000 years ago. Bedrock and surficial deposits are a complex mixture of granitic and gneissic rocks that provide limited acid buffering and a variety of less common metasedimentary formations with a range of acid buffering (Baker et al., 1990). Most soils in the region are classified as Spodosols. The mean annual precipitation ranges from approximately 800 to over 1600 mm across the region (Ito et al., 2002). Below-freezing temperatures occur through most of the winter, and with the onset of spring in late March or early April, accumulated snowmelts over several weeks cause the highest sustained stream flows of the year. The region is almost entirely forested with mixed northern hardwood and subboreal conifer species. Under the harsh climate and rugged terrain, historical agriculture was minimal, but logging occurred throughout the region and continues to date. However, there was little or no logging in the past two decades within the watersheds of any of the sampled streams.

This investigation included North and South Buck Creek monitoring watersheds (areas of 27 and 52 ha, respectively), located in the western Adirondack region of New York (NY) near the town of Inlet (Figure S1). North Buck has a wetland covered with a floating mat of shrub vegetation with some red spruce and speckled alder (*Alnus incana* (L.) Moench ssp. *rugosa*) near the top of the watershed covering 0.75 ha (2.8% of total watershed area). South Buck is well drained throughout without wetland influences. The two watersheds have mostly moderate slopes (average slope = 16% rise in both watersheds) and opposing aspects (North Buck facing south, South Buck facing north), with streams that drain into the main channel of Buck Creek approximately 250 m apart.

North Buck is forested by a mixture of hardwood and conifer species, whereas South Buck is dominated by beech (*Fagus grandifolia* Ehrh.) with only a small conifer presence in the overstory. In 2000, basal area of the North Buck forest was composed of 28% red spruce (*Picea rubens* Sarg.), 25% red maple (*Acer rubrum* L.), 22% beech, 10% hemlock (*Tsuga canadensis* L. Carr.), and 9.3% yellow birch (*Betula alleghaniensis* Britt.). Coniferous litter contributed to a thick forest floor (Oe plus Oa horizons) that averaged 21 cm in 1997. Basal area of South Buck was composed of 61% beech, 18% yellow birch, 15% sugar maple (*A. saccharum* Marsh.), and 1.7% red spruce and under the mostly hardwood canopy, the forest floor averaged 8 cm thick in 1998. Both watersheds are covered by mature undisturbed mixed-aged forests. No cutting has taken place for at least 50 years. Coring of canopy-dominant spruce trees in North Buck revealed trees ranging in age from 60 to >250 years. There were minimal changes in the relative abundances of species over the study.

### 2.2. Deposition and Climate Data

Annual mean concentration data in wet deposition for 2000–2015 were obtained from the National Atmospheric Deposition Program (NADP) collection Station NY20 (Huntington Wildlife) near Newcomb, NY, approximately 48 km northeast of Buck Creek. Wet deposition data and annual total deposition for S and N (TDEP) from 2000 to 2015 were obtained from the NADP website (http://nadp.slh.wisc.edu/committees/tdep/tdepmaps/; accessed 1 May 2017). The TDEP data were derived from wet and dry deposition monitoring data that were combined with spatial modeling to develop a 4-km^2^ gridded spatial coverage, as described in Schwede and Lear (2014). Simple linear regression was used for trend analysis of these data.

Spatially continuous estimates of mean monthly precipitation and air temperature were downloaded from the PRISM climate database (http://prism.oregonstate.edu/, accessed May 2017). Monthly areal mean estimates of air temperature and precipitation were extracted for the Adirondack ecological region using geographic information software, for 1983–2015. Annual areal mean temperature and precipitation were estimated by calculating the 12-month mean for a given year. Likewise, seasonal areal mean temperature and precipitation values were estimated by taking the mean of the months that fell within the respective seasons (as described above). Simple linear regression analysis was used to establish trends and to explore relationships with stream ${{\text{NO}}_{3}}^{-}$ export and concentrations.

### 2.3. Stream Water Monitoring and Analysis

Stream gages operated by the U.S. Geological Survey (USGS) at the base of North and South Buck Creek watersheds (USGS Stations 04253295 and 04253294, respectively) provided daily average flow, year-round, following methods of Rantz (1982). Data from these gaging stations are available from the USGS National Water Information System (NWIS) database (U.S. Geological Survey, National Water Information System —Web interface, accessed at https://doi.org/10.5066/F7P55KJN, 28 March 2019). At each stream gaging station, stream water was collected for chemical analysis one to three times per month from January 2000 to May 2001, either manually or with flow-activated autosamplers, except for February 2001 when samples were not collected. Samples thereafter were collected manually every 2 weeks from June 2001 to December 2015, except for occasions in August when North Buck was dry.

All stream samples were chemically analyzed for ${{\text{NO}}_{3}}^{-}$, ${{\text{NH}}_{4}}^{+}$, ${{\text{SO}}_{4}}^{2-}$, ${\text{Ca}}^{2+}$, DOC, pH, and total and organic forms of monomeric Al in either the USGS New York Water Science Center Soil and Low-Ionic Strength Water Quality Laboratory in Troy, NY (hereafter the USGS Troy Laboratory) or the Adirondack Lakes Survey Corporation laboratory in Ray Brook, NY. Inorganic monomeric Al (hereafter Al_i_) was determined by subtracting organic monomeric Al from total monomeric Al. Measurements of total dissolved nitrogen (TDN) were also made on a subset of samples in the USGS laboratory. Dissolved organic nitrogen (DON) was determined on these samples by subtracting ${{\text{NO}}_{3}}^{-}$ and ${{\text{NH}}_{4}}^{+}$ from the TDN measurements. A linear relationship between DOC and DON was then developed to enable DON to be estimated for the full study period. Complete details of the chemical analyses of North and South Buck Creek water samples are provided in the supporting information, including results of an interlaboratory comparison of results from the two laboratories (Figure S2). Monthly values of ${{\text{NO}}_{3}}^{-}$ concentrations in North and South Buck were determined by averaging biweekly values of the full record, which were then averaged for the periods 1999–2008 and 2009–2015. Possible differences in these periods were evaluated with *t* tests or Mann-Whitney rank sum tests where normality was rejected.

Effects of acidic deposition on Adirondack stream chemistry were previously evaluated in approximately 200 headwater streams (mostly first order) by the Western Adirondack Stream Survey (WASS) in the heavily acid rain impacted western Adirondack region in 2004–2005 (Lawrence et al., 2008a; Lawrence et al., 2008b). To support the objectives of this study, data collected once during primary spring snowmelt in 2004 and 2005 from 184 WASS streams were included (Figure S1). Also included were WASS data from 63 of these streams that were resampled during primary spring snowmelt in 2014 and 2015. For comparison, snowmelt data were averaged for each of the two periods (2004–2005 and 2014–2015). In addition, the analysis herein includes 56 streams sampled once during August base flow in 2004 and again during August base flow in 2014. Stream flow monitoring at Buck Creek was used to evaluate comparability of flow conditions on the different sampling dates. Additional details on the WASS sampling design and chemical analyses are provided in the supporting information. Paired *t* tests (or Wilcoxon rank sum tests where normality was rejected) were used to determine if there were differences between the two sampling periods for both snowmelt and August baseflow samplings.

The WASS sampling design was also used in the previously conducted East-Central Adirondack Stream Survey (ECASS), which included streams located throughout the Adirondack region not covered by WASS sampling (Figure S1). ECASS data were included in this paper from 198 streams sampled once during primary spring snowmelt in 2011. Further details of the ECASS sampling and analysis are provided in the supporting information and Lawrence, Roy, et al. (2018). For this analysis, ECASS data were combined with the 2004–2005 WASS snowmelt data to yield a total of 382 Adirondack streams located throughout the 24,000-km^2^ Adirondack region. Piecewise linear regression (two segments) was used to evaluate the relationship between ${{\text{NO}}_{3}}^{-}$ concentrations and the concentration ratio of ${\text{Ca}}^{2+}$ to ${{\text{SO}}_{4}}^{2-}$ in the combined WASS/ECASS data set.

All stream chemistry data used in this paper are available from the USGS NWIS database (U.S. Geological Survey, National Water Information System—Web interface, accessed at https://doi.org/10.5066/F7P55KJN, 28 March 2019). NWIS database codes and coordinates for all stream sampling locations are listed in Table S1.

### 2.4. Watershed Nitrogen Flux and Storage Calculations

The major pools and fluxes of N in North and South Buck were compared to address the question of why North and South Buck watersheds maintained elevated N export (kg N·ha^−1^·year^−1^) despite a declining trend in atmospheric deposition of N. As such, annual N deposition totaled for the years 2000–2015 was related to the sum of totaled annual N export in stream water plus the gain in tree biomass N for this period to provide insight into the role of the soil N pool. This information was then compared with measured changes in N content in the upper soil pool for the same period. Other known pools and fluxes were not included in this analysis because they were not essential to address this question and were difficult to estimate on a watershed basis without introducing large uncertainty.

For much of the data record, nontransformed relationships between concentrations of ${{\text{NO}}_{3}}^{-}$ (and ${{\text{NH}}_{4}}^{+}$) and flow were either nonexistent or weak and variable across seasons and years. Therefore, watershed export of dissolved inorganic N (DIN) was determined by calculating a monthly mean concentration for ${{\text{NO}}_{3}}^{-}$ and ${{\text{NH}}_{4}}^{+}$ from the two to three water samples collected in each month, which was then multiplied by the total daily flow to compute watershed dissolved inorganic N export as kilograms of nitrogen per hectare per day. This method of calculating watershed export values gave similar results to those presented in Sabo et al. (2016). Estimates of monthly mean DON concentrations were used in the same manner to determine watershed DON export. Daily values of total N export (${{\text{NO}}_{3}}^{-}$, ${{\text{NH}}_{4}}^{+}$, and DON) were then summed seasonally or annually. Trends in annual values (both yearly totals and seasonal totals) were evaluated with simple linear regression.

To evaluate the role of soil in watershed N retention and export, soil samples were collected within 15 m of 28 permanent point locations established in each watershed along seven transects distributed from upper to lower elevations, perpendicular to the fall line. Soil samples from the Oe, Oa, and upper 10 cm of the B horizons were collected from all these locations in 1997 in North Buck and 1998 in South Buck (see the supporting information). Horizon identifications were done according to Schoeneberger et al. (2012). Soil pits were excavated with shovels to expose a pit face that was used for measurement of horizon thickness and collection of soil. Sampling was repeated using the same methods at the same locations in North Buck in 2009–2010 and South Buck in 2014. The soil resampling procedures used in this study are further detailed in Lawrence, Fernandez, Hazlett, Bailey et al. (2016). All soil samples were analyzed in the USGS Troy Laboratory following U.S. Environmental Protection Agency (USEPA) methods (Blume et al., 1990) and Thomas (1982) as described in the supporting information. All soil chemical data are available as a USGS data release (Lawrence, Sullivan, Bailey, et al., 2017). Standard deviation values and coefficients of variation reflecting the spatial variability for all soil measurements in each watershed are listed in Tables S2 and S3.

The total horizon mass of N, C, and Ca was calculated from concentration values, horizon thickness measurements, and bulk density estimates determined by measurements of loss on ignition and the model of Federer et al. (1993). Coarse fraction of the B was estimated to be 14.4% of volume from an average of nine pits in watersheds near North and South Buck with highly similar soils. Coarse fraction was determined by excavating a measured volume of the entire B horizon (top to bottom) and weighing all fragments not passing through a 1.0-cm screen. Rock density was assumed to be 2,600 kg/m^3^.

Differences in measurements between the two sampling periods were evaluated by watershed (*n* = 28) with paired *t* tests based on the permanent sampling markers. The confidence limits of changes between the two sampling periods were determined for all changes where *P* < 0.05. Differences in measurements between the two watersheds were evaluated with unpaired *t* tests or Mann-Whitney rank sum tests where normality was rejected.

For vegetation measurements, circular plots with 9-m radius (254 m^2^) were centered on 15 of the permanent point locations in both North and South Buck, with one to three plots on each transect. All trees within the plots with diameter at breast height (DBH) of at least 5 cm were identified and measured for DBH at a fixed tag approximately 1.4 m from the ground in the growing seasons of 2000, 2005, 2010, and 2015. All vegetation measurements collected in North and South Buck plots are available as a USGS data release (Lawrence, McDonnell, & Sullivan 2017). Simple linear regression was used to evaluate trends from 2000 to 2015.

Estimates of complete tree (above and belowground) and foliage biomass were determined from DBH measurements and species-specific allometric equations from Young et al. (1980), summarized in Jenkins et al. (2004). Wood biomass was estimated for each tree by subtracting the foliage biomass from the complete tree biomass. Individual tree foliage, wood, and complete tree biomass estimates were summed by plot and then averaged to estimate mean plot-level biomass for each watershed. The mean plot-level biomass (biomass per hectare) was multiplied by the areas of the respective watersheds to estimate total foliage, wood, and complete tree biomass at 5-year intervals from 2000 to 2015. Additional information on biomass estimation is available in the supporting information. The individual tree biomass estimates were used to calculate wood (above and belowground) and foliage N using species-specific N concentration values (% N) for bole wood and foliage (Adams et al., 1995; NERC, 2010; Pardo et al., 2005). Species-specific N concentration data were not available for roots, and the wood biomass estimates were not compartmentalized into bark, bole, branches and roots, so bole wood N concentrations were used to estimate all wood N concentrations. Additional information on estimation of N stored in wood is available in the supporting information.

All statistical analyses in this study were done with SigmaPlot 13 or SAS 9.4.

## 3. Results

### 3.1. North and South Buck Watersheds

**Figure 1.**
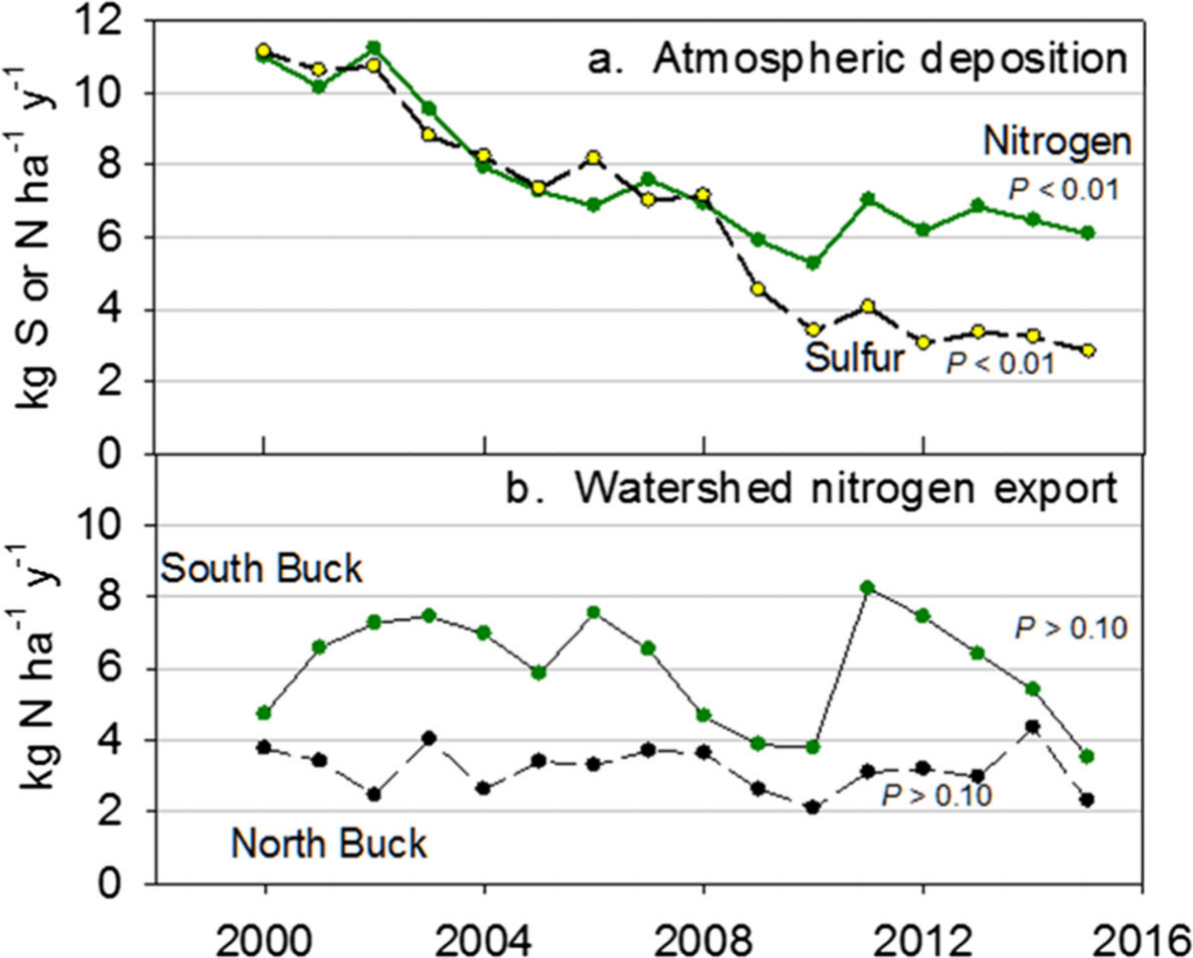
Annual values of (a) total atmospheric S and N deposition and (b) total dissolved N export in stream water from north and South Buck watersheds. Values of *P* indicate significance of a trend over time based on linear regression.

**Figure 2.**
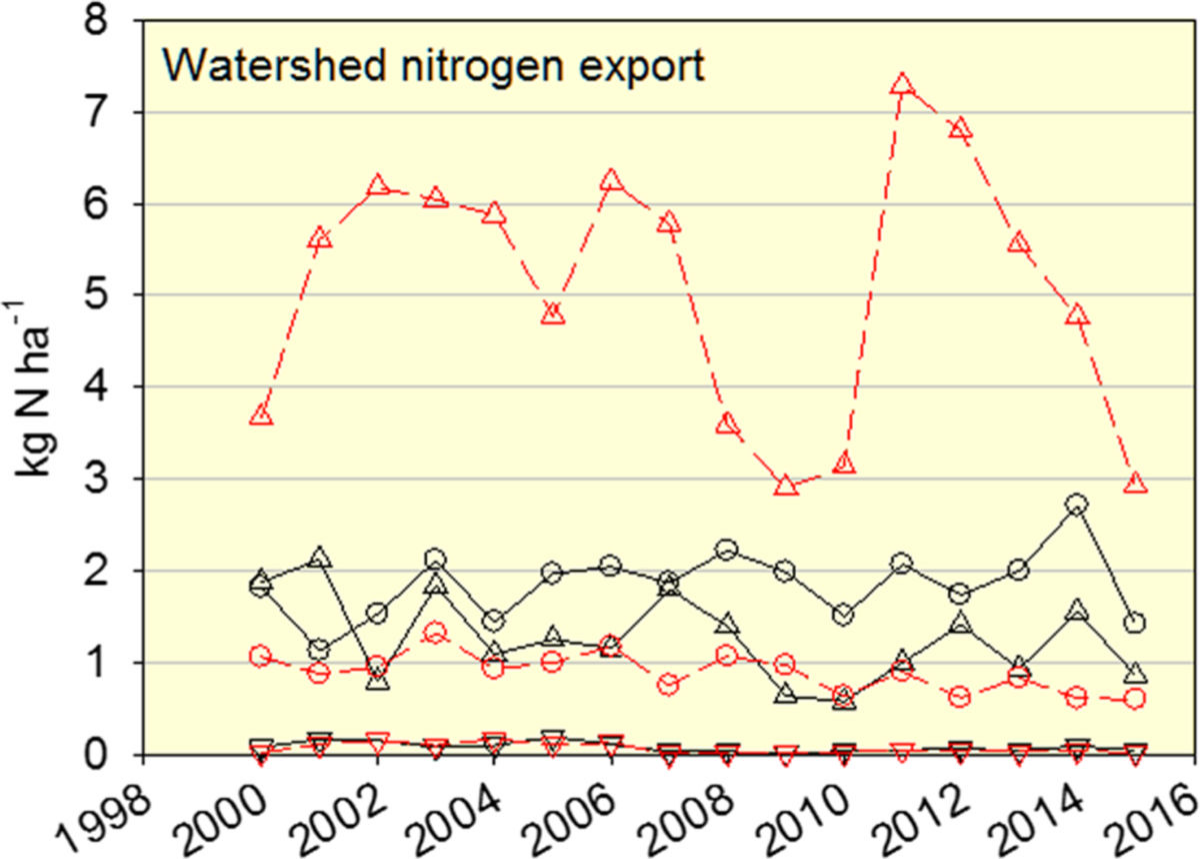
Annual values of watershed N export as ${{\text{NO}}_{3}}^{-}$ (triangles up), ${{\text{NH}}_{4}}^{+}$(triangles down), and organic N (circles) in stream water of north (black symbols and solid lines) and south (red symbols and dashed lines) Buck watersheds.

The total atmospheric deposition of S and N decreased 73% and 45%, respectively, from 2000 to 2015, although N showed no trend for 2008–2015 (Figure 1a). In contrast, annual watershed export of TDN did not show trends in either North or South Buck watersheds for 1999–2015 (Figure 1b). Values of N export were considerably higher in South Buck than North Buck, as were fluctuations among years. A clear difference in N export between watersheds was also found when N export was expressed by individual N species. In North Buck, export of organic N was similar to export of ${{\text{NO}}_{3}}^{-}$, although somewhat higher (Figure 2). This relationship differed greatly from South Buck, where export of ${{\text{NO}}_{3}}^{-}$ was substantially higher than organic N export (Figure 2). Export in the form of ${{\text{NH}}_{4}}^{+}$ was minimal in both watersheds, which makes the difference in the total N export between watersheds attributable to the high ${{\text{NO}}_{3}}^{-}$ export in South Buck. No trends in the export of any N species in either watershed were observed over the study period except for a significant (*P* = 0.073) decrease in ${{\text{NO}}_{3}}^{-}$ in North Buck.

**Figure 3.**
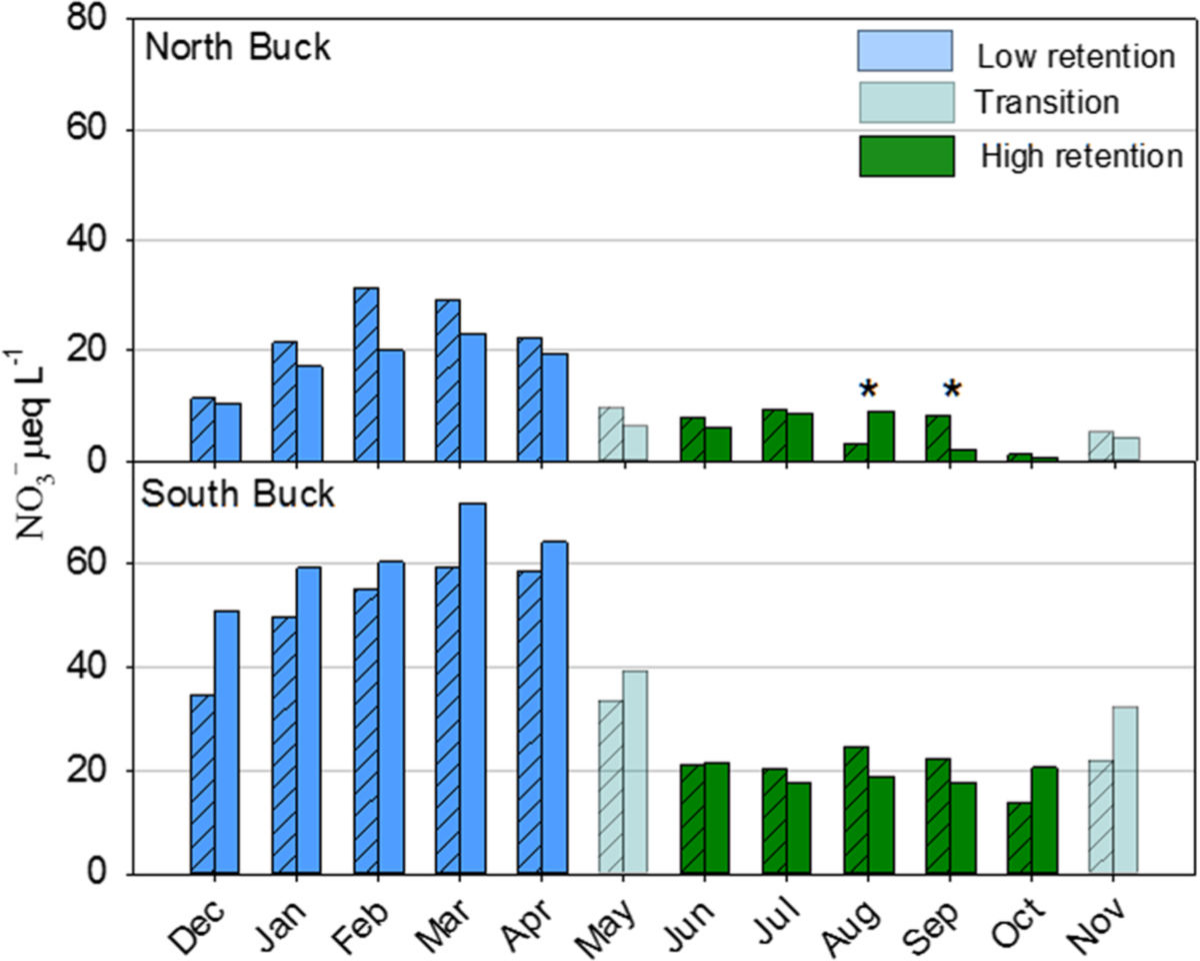
Monthly average concentrations of ${{\text{NO}}_{3}}^{-}$ in the streams of north and South Buck watersheds. Cross-hatched bars represent means of monthly values in each year from 1999 to 2008, a period of decreasing atmospheric N deposition. Unhatched bars represent means of monthly values in each year for 2009–2015, a period with no trend in atmospheric N deposition. Colors represent the months when watershed export of N are highest (blue, December–April), lowest (dark green, June–October), or in transition (cyan, May and November). Significant differences (*P* < 0.05) between the same months of different deposition periods are indicated by asterisks. The mean of all months in North Buck was higher (*P* < 0.05) for 1999–2008 than for 2009–2015, whereas in South Buck, this value was lower (*P* < 0.05) for 1999–2008 than 2009–2015.

Concentrations of ${{\text{NO}}_{3}}^{-}$, the primary form of dissolved inorganic N in the streams draining the North and South Buck watersheds, showed a seasonal pattern that was highly consistent from year to year in both watersheds. Highest concentrations were measured from December through April, whereas lowest concentrations were measured in June through October (Figure 3). The lowest ${{\text{NO}}_{3}}^{-}$ concentrations tended to occur in October, outside of the growing season of this region (Figure 3). On this basis, watershed retention of N was characterized in terms of high and low retention seasons rather than growing and nongrowing seasons, as is typically done. May and November serve as transitional months as forest ecosystems undergo the changes needed to shift from dormancy to growth and vice versa. Stream ${{\text{NO}}_{3}}^{-}$ concentrations differed considerably between watersheds for all individual months (*P* < 0.05). Also, in North Buck, mean ${{\text{NO}}_{3}}^{-}$ concentrations for 2009–2015 (low deposition, no deposition trend) were lower than for 1999–2008 (high deposition, decreasing deposition trend). However, in South Buck, stream ${{\text{NO}}_{3}}^{-}$ concentrations were lower for 1999–2008 than 2009–2015, which was the reverse of what was seen in the N deposition. When separated by low and high retention season, watershed N export exhibited no trends for 2000–2015 in either season for either watershed (Figure S3).

**Figure 4.**
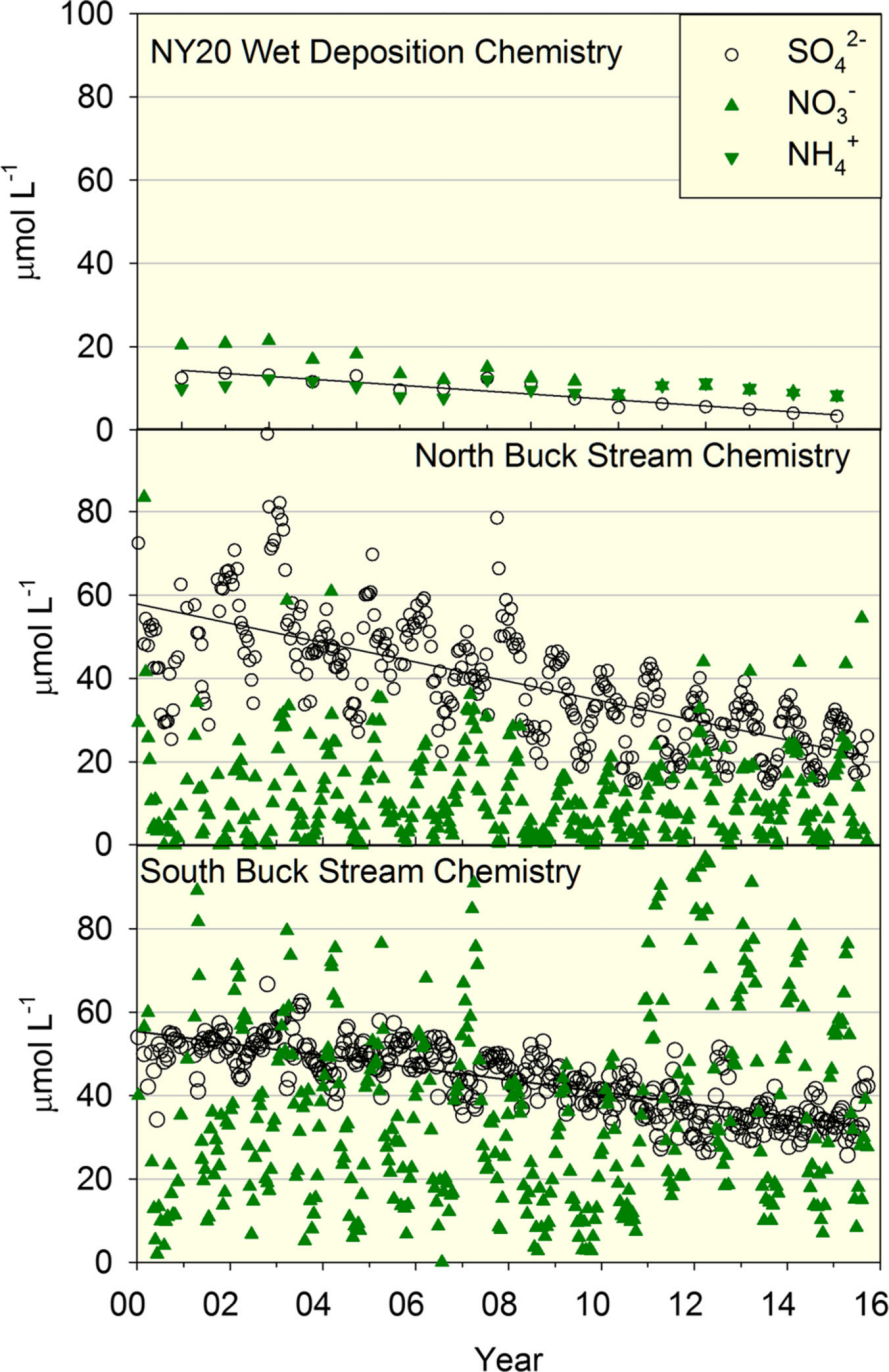
Annual mean volume-weighted concentrations of ${{\text{SO}}_{4}}^{2-}$, ${{\text{NO}}_{3}}^{-}$, and ${{\text{NH}}_{4}}^{+}$ in wet deposition measured at National Atmospheric Deposition Program station NY20 and biweekly stream concentrations of ${{\text{SO}}_{4}}^{2-}$ and ${{\text{NO}}_{3}}^{-}$ in north and South Buck from 2000 to 2015. Best fit lines (*P* < 0.01) are shown for concentrations of ${{\text{SO}}_{4}}^{2-}$ in wet deposition and stream chemistry in both watersheds.

As with S deposition, annual mean concentrations of ${{\text{SO}}_{4}}^{2-}$ in wet deposition showed a clear decreasing trend (*P* < 0.01) throughout the study period (Figure 4). A strong decreasing trend in ${{\text{SO}}_{4}}^{2-}$ concentrations (*P* < 0.01) also occurred in both streams (Figure 4), but stream concentrations were much higher than in wet deposition throughout the study period, which indicated that watershed processes were substantially increasing ${{\text{SO}}_{4}}^{2-}$ concentrations in drainage waters. Concentrations of ${{\text{NO}}_{3}}^{-}$ also decreased in wet deposition, whereas concentrations in North Buck did not exhibit a trend (*P* > 0.10) and were highly variable, exhibiting values both above and below wet deposition concentrations (Figure 4). Concentrations of ${{\text{NO}}_{3}}^{-}$ in South Buck were also highly variable but did increase over the study period (*P* < 0.01) and were generally were much higher than wet deposition concentrations (Figure 4). Concentrations of ${{\text{NH}}_{4}}^{+}$ in wet deposition did not exhibit a trend over the study period (*P* > 0.10) and were extremely low in the streams of both watersheds (mean of study period = 0.9 μmol/L).

**Figure 5.**
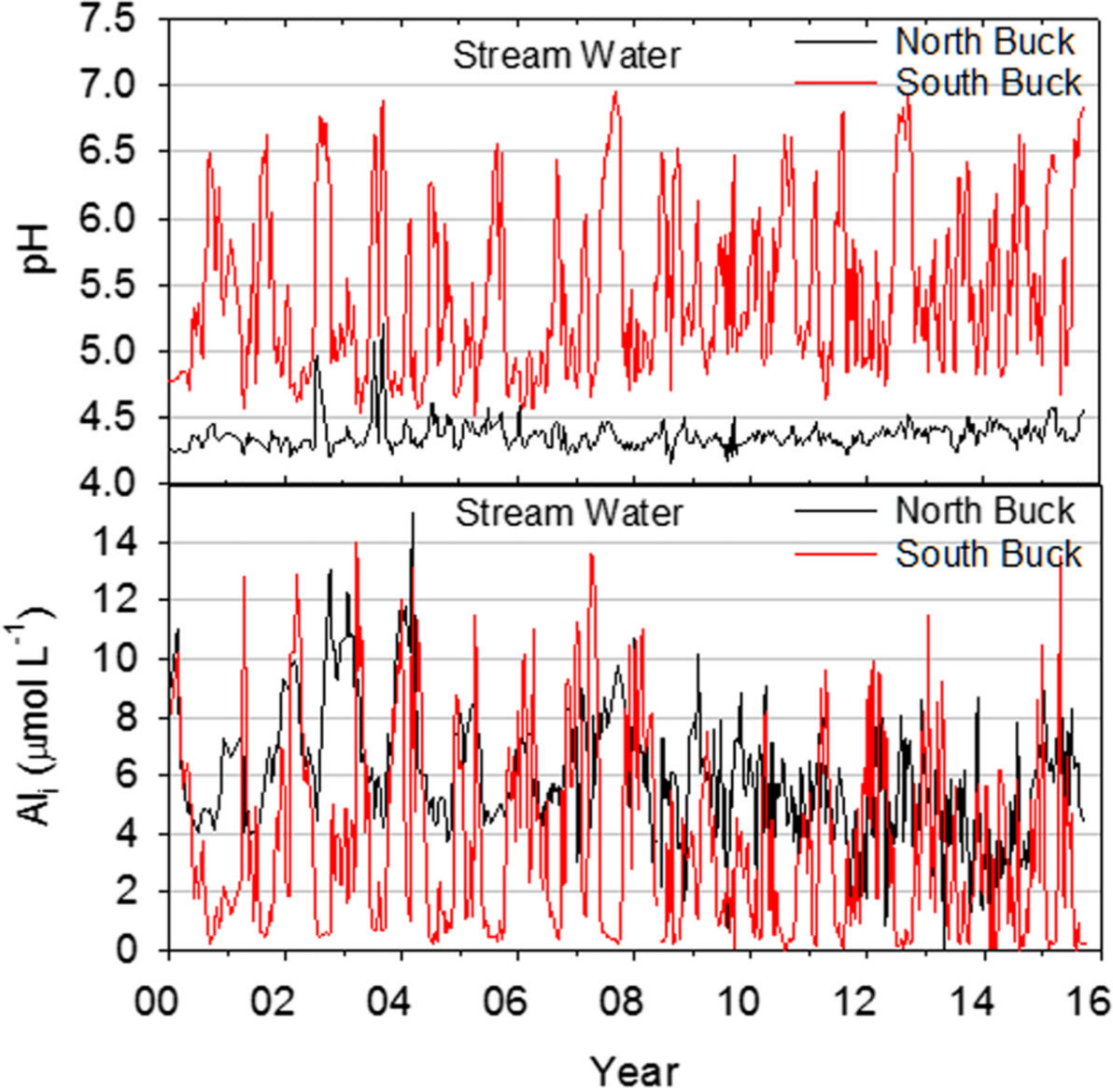
Values of pH and Al_i_ (inorganic monomeric Al) concentrations in north and South Buck stream water during the study period.

Stream chemistry also showed distinct differences in pH and concentrations of inorganic monomeric Al between watersheds. North Buck pH values remained consistently below 4.5 for nearly the entire study period, whereas South Buck pH values fluctuated widely from near 4.5 to above 6.5 (Figure 5). In North Buck, pH increased over the study period (*P* < 0.05) but the stream remained extremely acidic due to high concentrations of organic acids. In South Buck, pH showed a stronger increase over the study period (*P* < 0.01), primarily due to an increase in minimum levels, but values below 5.0 continued to be common throughout the record. Concentrations of Al_i_, however, were similar between watersheds, although minimum values rarely decreased below 4.0 in North Buck but seasonally decreased below 1.0 μmol/L in South Buck (Figure 5). These concentrations of Al represent extremely harmful conditions for fish communities (Baldigo et al., 2019).

**Figure 6.**
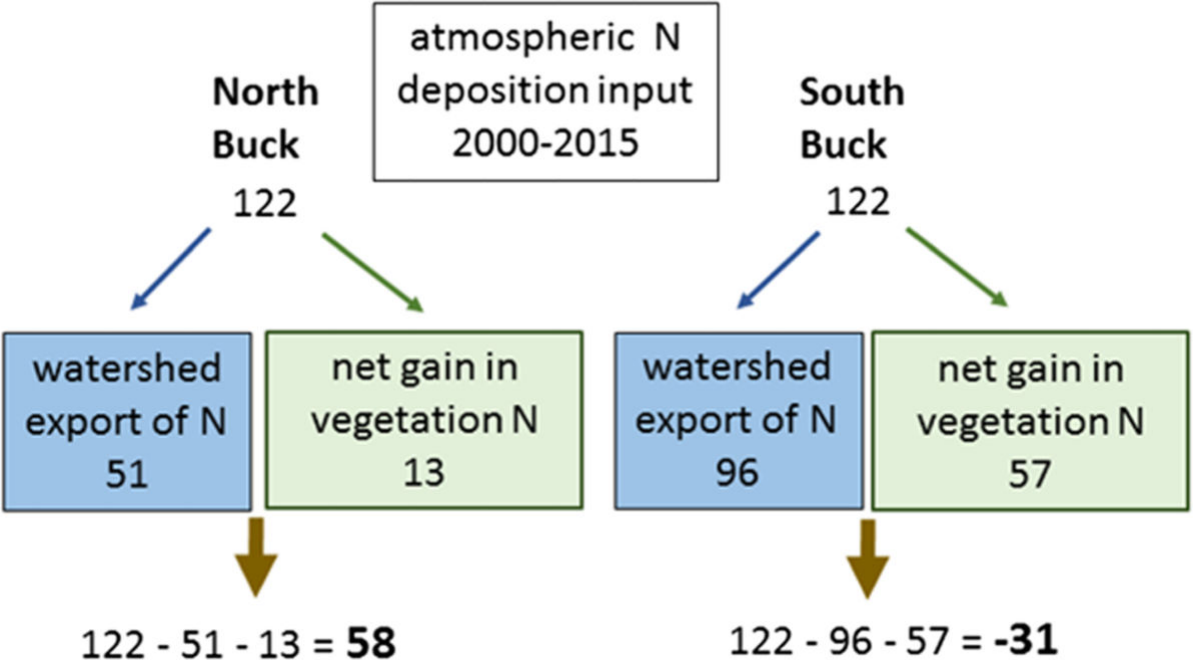
Annual N deposition totaled for the years 2000–2015 compared to the sum of N export in stream water plus the gain in tree biomass N for this period. Subtraction of watershed export and net vegetation growth from deposition (shown in bold) suggests that North Buck soils act as a sink for N deposition, whereas South Buck soils act as a source of stream water N. All values are in kilograms per hectare.

Tree basal area, tree biomass, and foliar biomass in South Buck increased in each of the intervals between the 5-year measurements and over the full study period (*P* < 0.05) but did not increase (*P* > 0.10) for any of the intervals or overall in North Buck (Table 1). Storage of N in both foliage and wood increased in South Buck from 2000 to 2015 (*P* < 0.05), whereas neither measurement increased in North Buck (*P* > 0.10), although there was some indication of an increase in wood N (*P* < 0.10). Relating N deposition to watershed export and tree N retention revealed a marked contrast between watersheds. For the period 2000–2015, watershed export of N plus the gain in tree N storage (64 kg/ha) in North Buck could account for only 48% of the atmospherically deposited N (122 kg/ha), whereas in South Buck, the sum of these factors (153 kg/ha) exceeded the full atmospheric input by 25% (Figure 6). This analysis suggests that in the absence of high gaseous N losses in North Buck, soils acted as a net sink for N deposition, whereas in South Buck, soils acted as a net source of N.

Comparison of soils between watersheds in the initial sampling (North Buck 1997; South Buck 1998) showed distinct differences indicated by pink and red shading in the South Buck (1998) column of Table 2. Organic C concentration (g/kg) and content (Mg/ha) were higher in North Buck than in South Buck in all three sampled horizons. Values of effective cation exchange capacity (CEC) were also higher in North Buck than South Buck in the Oa and upper B. Base saturation in the Oe was higher in North Buck than South Buck as well, but, in the Oa, was lower in North Buck than South Buck and did not differ in the B. Markedly higher acidity in North Buck than South Buck was indicated by higher H and lower pH in all three horizons. Values of Al saturation and Al:C were considerably lower in North Buck than South Buck in the Oe, whereas Al:C was higher in the Oa in North Buck than South Buck. Concentrations of N were higher in North Buck than South Buck in the B, as were N content in the Oa. Values of C:N were higher in North Buck than South Buck in all three horizons.

Significant changes in soils occurred between the initial and final samplings, indicated by asterisks in Table 2. These changes increased the dissimilarities between the two watersheds over time (Table 2, indicated by greater red shading for 2014 than 1998) particularly in the Oe. In the Oe of North Buck, organic C concentration and content increased, whereas in the Oe of South Buck, organic C concentration did not change, and C content decreased. In the Oe of North Buck, increases in CEC and pH, and decreases in Al saturation and Al:C, indicated that acidification had decreased, although H in the Oe increased. In South Buck, a large increase in base saturation and large decreases in H, Al saturation, and Al:C indicated that the decrease in acidification was stronger than in North Buck. These changes increased the difference in Oe acidification levels between North and South Buck relative to the initial sampling.

Resampling of the Oa revealed fewer changes in mean values than in the Oe. However, increases in concentrations of organic C and N and a decrease in Al:C were measured in the Oa of North Buck, and decreases in CEC and Al:C were measured in the Oa of South Buck (Table 2). Decreases in C and N content in the South Buck Oa may also have occurred but were marginally significant (*P* = 0.090). The upper B horizon in North and South Buck indicated a similar overall increase in acidity in both watersheds (Table 2), although pH and H did not change over time in the Oa or B of either watershed. Values of CEC in this horizon increased in both watersheds, but base saturation decreased in both watersheds largely due to increases in Al, which were reflected in increases of Al saturation and Al:C measurements in both watersheds. Organic C content also decreased in the B horizon of South Buck, as did N content to a greater extent, which resulted in an increase in C:N.

**Figure 7.**
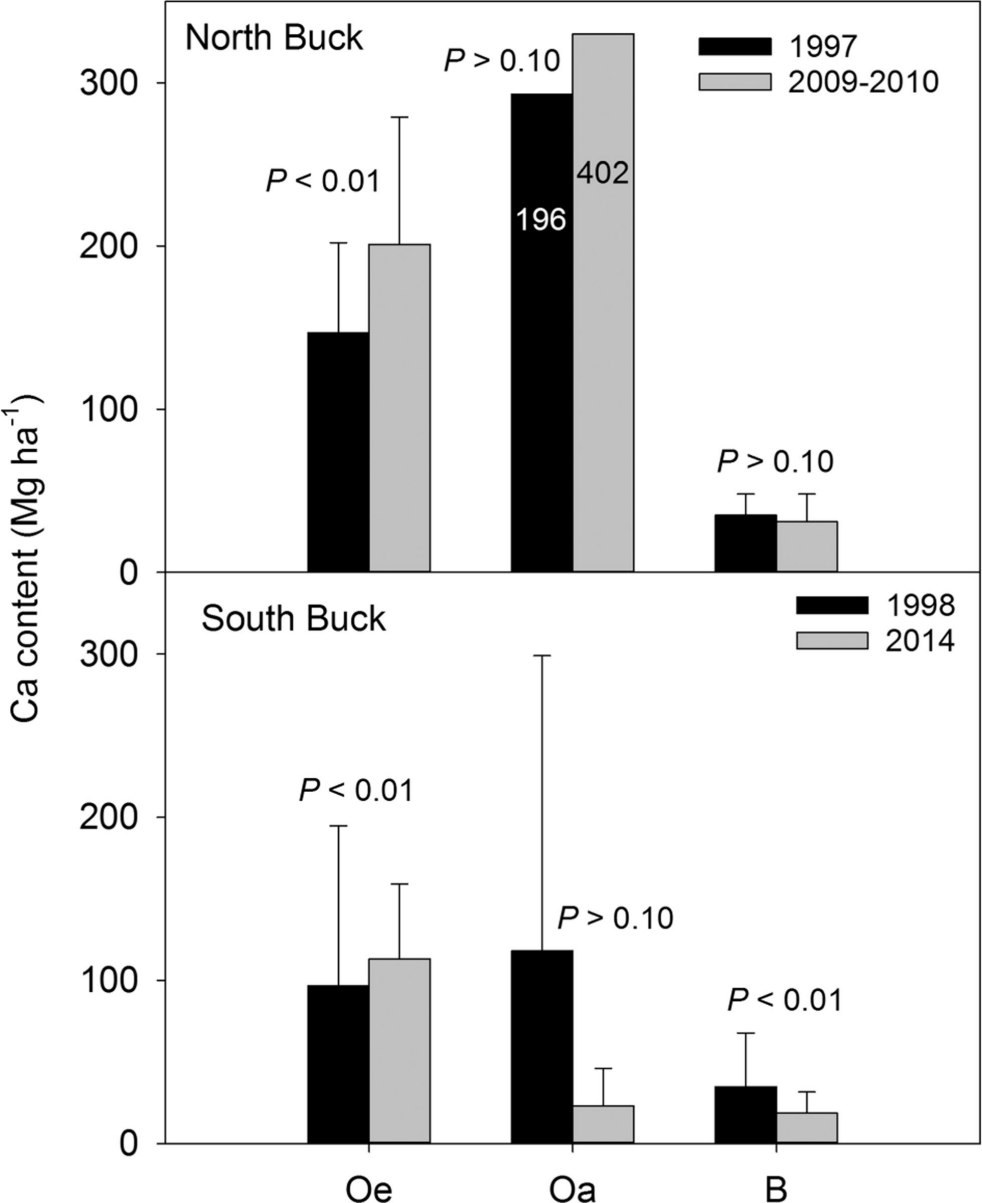
Mean values of Ca content in north and South Buck watersheds measured in initial and final samplings in the Oe, Oa, and upper 10 cm of the B horizons. Capped vertical lines indicate standard deviation values except for the North Buck Oa horizon where they are shown numerically within the bars.

To further evaluate the differences in the changes of soil acidity, soil Ca content (Mg/ha) was compared between watersheds (Figure 7). In North Buck, Ca content increased between samplings in the Oe horizon, but changes were not detected in the Oa or upper B horizon. In South Buck, Ca content also increased in the Oe horizon and decreased in the upper B horizon, but a significant change (*P* < 0.10) was not detected in the Oa horizon despite a 63% decrease in mean values. The lack of statistical difference was due to the large standard deviation of the Ca content values in 1998 (181), which was 1.5 times as large as the mean, whereas in 2014, the standard deviation (23.3) was approximately half of the mean. A paired *t* test was not used because normality of the 1998 data was disproven. Although a large difference occurred between means, median values for 1998 and 2014 were nearly identical (40.3 and 40.7 for 1998 and 2014, respectively). Therefore, no difference was found with the Wilcoxon rank test.

Trends in temperature and precipitation were also considered as possible factors influencing watershed N loss in North and South Buck. Regional PRISM data did not show significant trends (*P* > 0.10) in either temperature or precipitation over the 17-year study period for either the high or low retention season. There were also no correlations between temperature and N export for either high or low retention seasons in either watershed (*P* > 0.10), and no correlations between precipitation and N export for the low retention season in either watershed or for the high retention season in North Buck. However, there was a positive correlation (*r* = 0.52) between precipitation and N export during the high retention season in South Buck (*P* < 0.05), although stream flow did not show a trend during the high retention period (*P* > 0.10) in either watershed.

### 3.2. Regional Assessments

**Figure 8.**
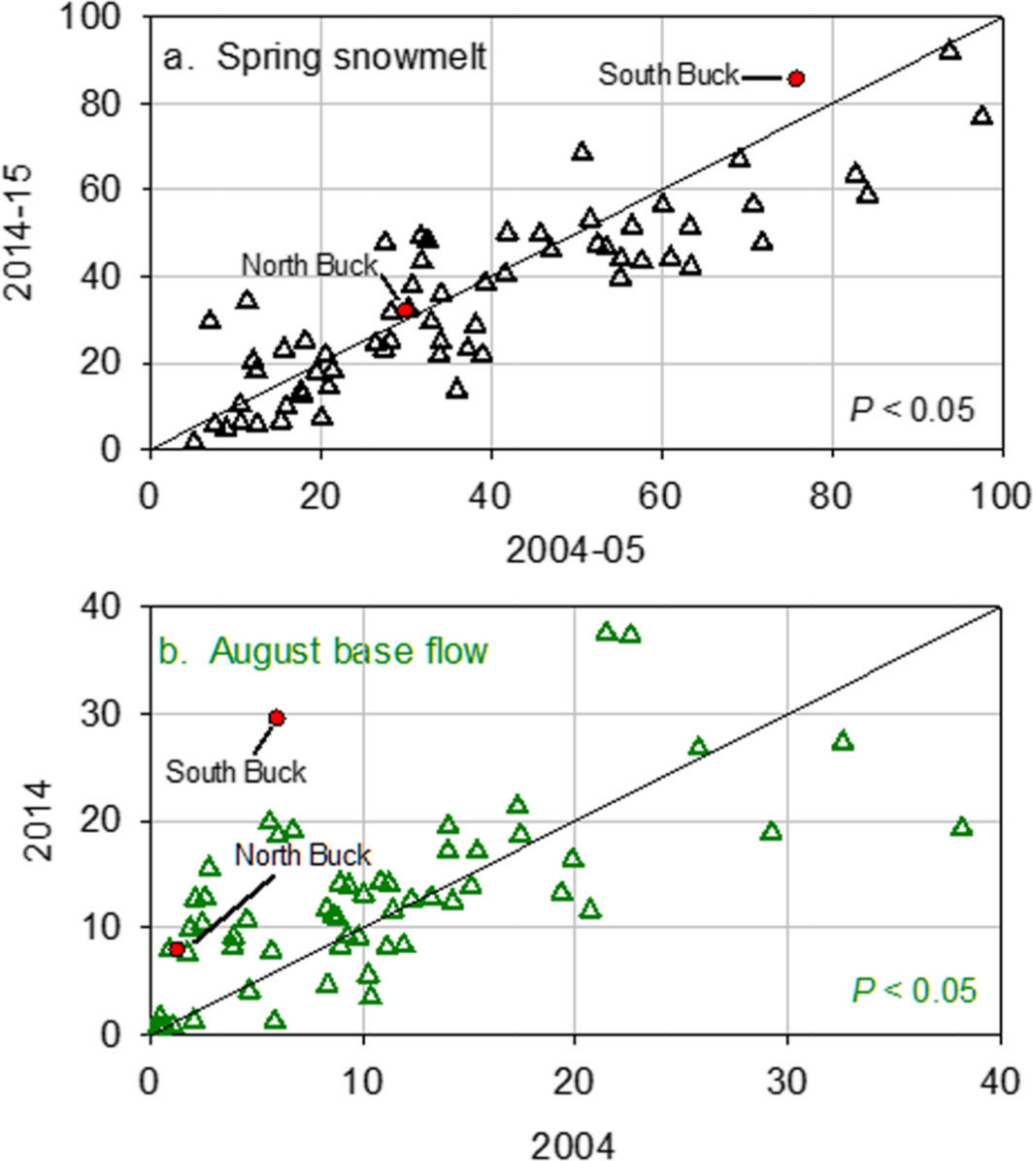
(a) Mean ${{\text{NO}}_{3}}^{-}$ concentrations in 63 Western Adirondack stream survey streams (triangles) sampled during spring snowmelt in 2004 and 2005 versus the mean of ${{\text{NO}}_{3}}^{-}$ concentrations in the same streams sampled during spring snowmelt in 2014 and 2015; (b) stream ${{\text{NO}}_{3}}^{-}$ concentrations in 56 Western Adirondack stream survey streams (triangles) sampled during august base flow in 2004 versus ${{\text{NO}}_{3}}^{-}$ concentrations in the same streams sampled during august base flow in 2014. Values for north and South Buck within 7 days of the stream surveys are shown as red-filled circles.

Surveys of 63 streams conducted in 2004–2005 and 2014–2015 indicated changes in ${{\text{NO}}_{3}}^{-}$ concentrations over time that varied depending on the season of sampling. During primary snowmelt, individual streams showed both increases and decreases between 2004–2005 and 2014–2015 (Figure 8a) but, as a group, showed a decrease in the overall mean concentration from 39 to 34 meq/L (*P* < 0.05). Percentiles of daily average flows during the 2004–2005 samplings ranged from 88.5 to 98.6 (the percent of days with less flow for calendar years 2002–2017) and 90.0 to 99.8 for the 2014–2015 samplings. During August base flow, the concentrations were considerably lower than during snowmelt (Figure 8b), but the overall mean increased from 10 μeq/L in 2004 to 12 meq/L in 2014 (*P* < 0.05). Percentiles of daily average flows during the August 2004–2005 samplings ranged from 29.0 to 46.4 and were 8.0 on each day of the 2014–2015 samplings. Although the flows during August samplings differed considerably between sampling periods, the ${{\text{NO}}_{3}}^{-}$ concentrations were similar. The means of combined April snowmelt and August baseflow data for the stream surveys were not statistically different (*P* > 0.10) between 2004–2005 and 2014–2015. The mean ${{\text{NO}}_{3}}^{-}$ concentrations of the two Buck watersheds in April (Figure 3) bracketed much of the range across the group of survey streams during spring snowmelt (Figure 8a), with the North Buck value falling close to the 1:1 line and the South Buck value falling somewhat above the 1:1 line. During August base flow, the North Buck value fell in the lowest part of the range, somewhat above the 1:1 line (Figure 8b), and the South Buck value fell well above the 1:1 line with a midrange value measured in 2004–2005, but one of the highest values measured in 2014.

**Figure 9.**
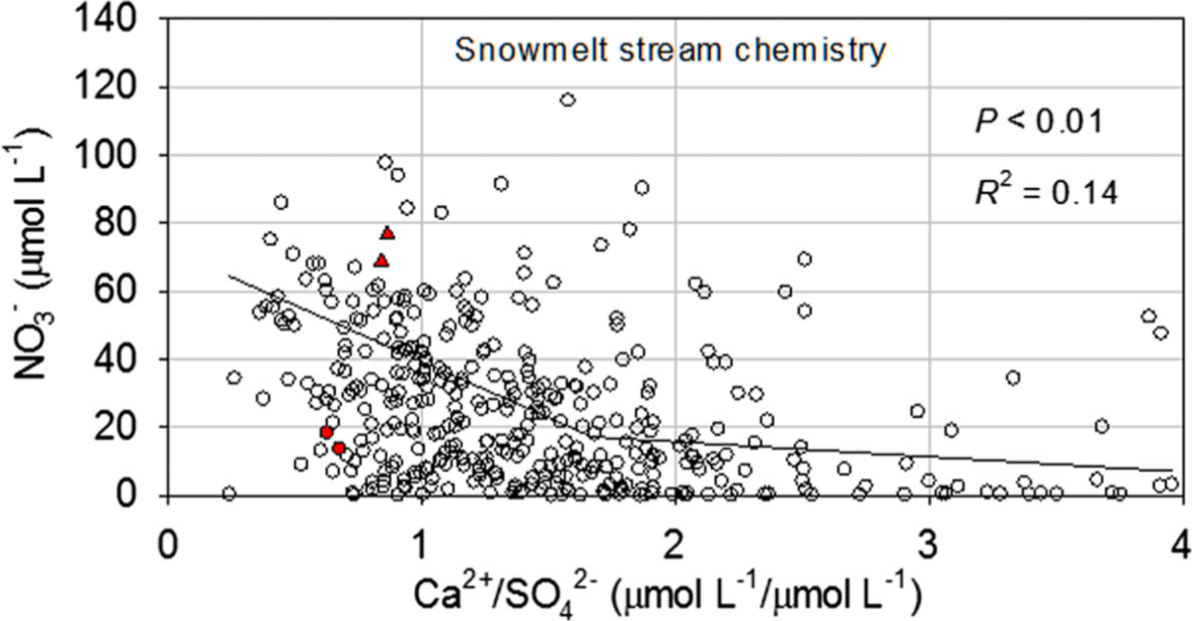
Concentrations of ${{\text{NO}}_{3}}^{-}$ as a function of the ratio of ${\text{Ca}}^{2+}$ to ${{\text{SO}}_{4}}^{2-}$ in streams sampled in 2004–2005 and 2011 throughout the Adirondack region. Values for North Buck (red circles) and South Buck (red triangles) are shown for snowmelt samples collected in 2004 and 2011. Piecewise regression showed a breakpoint at a ratio value of 1.67. *P* and *R*^2^ values represent the two-segment best fit line.

To evaluate how Ca availability related to watershed N loss across the entire Adirondack region, ${{\text{NO}}_{3}}^{-}$ concentrations from 414 WASS and ECASS streams sampled during snowmelt in 2004–2005 and 2011 were compared to the ratio of ${\text{Ca}}^{2+}$ to ${{\text{SO}}_{4}}^{2-}$ (${\text{Ca}}^{2+}$/${{\text{SO}}_{4}}^{2-}$) in stream water. This ratio provides an index of the capacity of watersheds to buffer atmospheric inputs of ${{\text{SO}}_{4}}^{2-}$ through the release of Ca from the soil and subsoil (Lawrence et al., 2011). The greater the value of the ratio, the greater the availability of Ca within the watershed. Depletion of Ca through acidic deposition decreases the value of the ratio. A negative correlation was observed between this ratio and ${{\text{NO}}_{3}}^{-}$ concentrations (Figure 9) in this large sample of streams. Piecewise regression indicated a significant relationship (*P* < 0.01) based on two line segments with a breakpoint at a Ca/${{\text{SO}}_{4}}^{2-}$ value of 1.67.

## 4. Discussion

### 4.1. Legacy Effects of Acidic Deposition on Soils

The soils of North and South Buck watersheds differed in whether they retained some of the N deposition or acted as a source of stream water N, but neither exhibited trends in stream export of N. Had the amount of N deposition that was retained within South Buck watershed (deposition-stream export) in 2000 (6.25 kg/ha) been replicated in 2015, all the N deposition for that year (6.1 kg/ha) would have been retained within the watershed. Instead, 3.53 kg/ha was exported in stream water in 2015, which indicated a marked reduction in the capacity of South Buck to retain N over the study period. No trend in N export in North Buck also suggested some reduction in the capacity to retain N deposition even though the watershed was retaining a substantial fraction. This result cannot be attributed to forest decline because the forests in both watersheds showed net growth over the study period. However, this result can be explained by a legacy effect of acidic deposition within the upper soil profile. As discussed in other studies (Evans et al., 2012; Monteith et al., 2007), past decades of acidic deposition are likely to have caused an increase in an accumulation of C and N in these soils through factors that included reduced organic matter solubility from decreased pH and increased ionic strength. Acidic deposition may have also enhanced C and N accumulation by lowering the rate of decomposition from decreased pH (Blagodatskaya & Anderson, 1998) and buildup of Al in the forest floor (Kunito et al., 2016; Scheel et al., 2007), as discussed in Oulehle et al. (2011). A proposed sequence of how these changes in acidic deposition affected soil N dynamics is summarized in Table 3.

Soil information predating the period of high acidic deposition is rare, but 54 sites throughout the Adirondack region sampled in 1932 and 1984 showed an increase (*P* < 0.05) in median organic matter content in the 0- to 10- and 10- to 20-cm layers of the mineral soil and a nonsignificant increase (*P* > 0.05) in the combined Oe/Oa horizons (Bedison et al., 2010). The authors attributed the accumulation largely to increased organic matter inputs from regrowth following logging in the late nineteenth and early twentieth centuries. However, these sampling dates also spanned the period of highest acidic deposition levels (Fakhraei et al., 2014). Although the primary mechanism of the twentieth-century C accumulation at these sites is not known, acidic deposition may have contributed to enhanced C and N retention. Consistent increases in C and N content of the top organic layer at the heavily acidified Solling, Germany, site were measured in a spruce stand (*Picea abies* L. Karst), from the mid-1960s through the late 1980s, but the increase leveled off through the 1990s (Meiwes et al., 2009). A consistent increase in C and N content was also measured in a beech stand (*Fagus sylvatica* L.) at Solling over a similar period, although the increase was considerably less than that of the spruce stand, and a subsequent decrease was suggested from 1993 to 2001. The increases in C and N content were interpreted to be largely the result of high deposition levels experienced at this site from the 1960s through the 1980s (Meiwes et al., 2009), and the contrast in responses between spruce and beech stands were highly similar to those in North and South Buck.

### 4.2. Nitrogen Fluxes in North and South Buck Watershed, 2000 to 2015

Hindcast deposition modeling suggests that by 2010, S deposition in the Adirondack region had decreased to levels similar to those in the early 1900s (Fakhraei et al., 2014). It is therefore likely that acidic deposition during the study period had less influence in enhancing accumulation of organic C in the Adirondack region than in previous decades. The Buck Creek soil data were largely consistent with this premise, with only an increase in C concentration measured in the Oe horizon of North Buck and only decreases or no change measured in South Buck. Nevertheless, in North Buck, the retention of N as a percent of deposition remained high, and ${{\text{NO}}_{3}}^{-}$ concentrations in stream water remained moderately low. Denitrification in the wetland near the top of the North Buck watershed was likely to have resulted in some loss of gaseous N that was not measured. However, the size of the wetland (2.8% of the watershed) and its position high in the watershed suggest a relatively small effect on watershed N loss relative to stream export. Direct flux of N to the atmosphere would mean that watershed N retention as a percent of atmospheric N was overestimated to some degree by our approach.

The high soil acidity naturally derived from coniferous litter in North Buck is largely responsible for the strong buffering of low pH values in stream water, in sharp contrast to South Buck, where pH values were higher, and variations were much greater. Soil organic acidity also helped to maintain low decomposition rates despite the decreased levels of acidic deposition (Blagodatskaya & Anderson, 1998). The high carbon concentration and content and C:N ratios in North Buck, which are typical of conifer-influenced soils in this region (Ross et al., 2009; Ross et al., 2011), also suggested moderate to low nitrification rates, which limit N release to surface waters, as summarized in the right column of Table 3. This interpretation is supported by the similarity in export of ${{\text{NO}}_{3}}^{-}$ and organic N in North Buck. Measurements from an earlier study in North Buck fell in the upper range of C:N ratios and the lower range of nitrification rates and watershed N export when compared with other northeastern watersheds (Ross et al., 2012).

Fluxes of N in South Buck differed markedly from those in North Buck. Substantial watershed N export occurred in South Buck despite the strong tree biomass sink relative to North Buck. Furthermore, the South Buck decrease in N content between soil samplings in the upper horizons far exceeded the difference between atmospheric inputs and the combined fluxes of tree uptake and watershed export over the study period. These findings pointed to the South Buck soil as a source of N rather than a sink, rarely reported in non-nitrogen-fixing forests experiencing net growth and contrary to what has been typically reported in the past in many watershed N studies. The sustained high export of N largely in the form of ${{\text{NO}}_{3}}^{-}$ under decreasing rates of atmospheric N deposition could have resulted from an increase in decomposition rates in the forest floor—a reversal of past organic matter accumulation that occurred under high deposition rates. This explanation is supported by the large decreases in atmospheric inputs of acidity and forest floor Al that can be linked to the decrease in C and N content through their stimulatory effect on decomposition rates. A large decrease in the ionic strength of precipitation may also have contributed to increased solubilization of organic matter (Hruska et al., 2009) that could enhance decomposition. Nitrogen fixation was unlikely to play a large role in N release in South Buck watershed or elsewhere in the Adirondack region because the only common N-fixing tree species (speckled alder) is restricted to wetlands, where denitrification processes can also occur (Mitchell et al., 2003).

The difference in watershed export of N in the streams of North and South Buck suggested a restraint on decomposition in North Buck relative to South Buck. Forest floor decomposition in North Buck appears to have been less responsive to decreases in acidic deposition due to the inherent effects of recalcitrant conifer litter that lowers pH and base saturation and increases exchangeable acidity. This interpretation is supported by higher organic N as a fraction of total N in stream water concentrations in North Buck than South Buck. However, this difference between watersheds may also be related to more shallow soil and subsoil in North Buck than South Buck. This factor would suggest that organic N would be more effectively retained in South Buck than North Buck because dissolved organic N mobility is limited in mineral soil, whereas ${{\text{NO}}_{3}}^{-}$ tends to be mobile throughout the soil and subsoil. The difference in soil chemical conditions between watersheds was also likely to have suppressed nitrification rates and therefore ${{\text{NO}}_{3}}^{-}$ concentrations in stream water in North Buck relative to South Buck, as measured by Ross et al. (2012). Organic functional groups associated with the much higher organic matter concentration and content in North Buck than South Buck also provided a greater capacity in North Buck to buffer against decreased acidity of soil solutions (Ross et al., 1996) as acidic deposition rates decreased. This interpretation is supported by a recent experiment that showed a more stressed microbial community in acidic soil of a Norway spruce stand than in soil of a less acidic beech stand at the start of the experiment, an effect that was intensified with the addition of sulfuric acid (Oulehle et al., 2018).

Much of the N and C released from decomposition in South Buck was likely to have been retained deeper in the soil profile, as documented elsewhere (Oulehle et al., 2011), but a consequential amount of the N released from the upper soil reached the stream channel in the form of ${{\text{NO}}_{3}}^{-}$, which supports the interpretation of nitrification sustained by enhanced N mineralization. Because stream export of N was based on fixed-interval sampling, the highest stream N concentrations, which are typically associated with the highest flows, were not incorporated into our export estimates. Watershed N exports in stream water were therefore underestimated to some degree. Soil denitrification, which was not measured in these watersheds, was also likely to have contributed to watershed N export but, in the well-drained South Buck watershed, was probably not a large contributor relative to the N export in stream water, based on estimates for similar watersheds in the region (Morse et al., 2015).

The soil changes occurring in South Buck would be considered favorable for tree growth, as described in Lawrence, McDonnell, et al. (2018), and were consistent with the trends of increasing tree and foliage biomass measured in South Buck but very unfavorable to stream ecosystems due to continued elevated Al_i_ leaching. Increased decomposition rates from lower acidic deposition would be expected to increase nutrient availability, and the high watershed N export suggested that N availability exceeded demand over the study period. Sabo et al. (2016) observed declining d^15^N values that suggested lower N availability before decreases in N deposition occurred in these watersheds(before 1995). The cause of this relationship is unclear but could perhaps be tied to decreases in S deposition that improved tree growth but had not yet enhanced forest floor mineralization

### 4.3. Tree Microbial Climate Interactions

Seasonal variation in watershed N retention and release is, in large part, a result of growing season plant demand. However, leaf drop during September and October provides an opportunity for microbial populations to play an important role in the seasonality of N dynamics. The large fall input of labile C with high C:N leads to N immobilization and minimum ${{\text{NO}}_{3}}^{-}$ concentrations in streams as microbial populations rapidly expand, as has been documented in other northeastern streams and forest floor soil solutions (Goodale et al., 2009; Sebestyen et al., 2014). As the pool of labile C is consumed through the fall and winter, heterotrophic populations peak and then begin to shrink without additional fresh C input. Availability of N then begins to increase through cell mortality of the shrinking heterotroph population, which provides low C:N substrate that favors autotrophic nitrifiers (Schimel & Bennett, 2004).

The lower snowmelt ${{\text{NO}}_{3}}^{-}$ concentrations seen in the 2014–2015 survey than in the 2004–2005 survey may have been related to differences in soil C availability in the nongrowing seasons of these years. Effects of differing hydrologic conditions and soil frost regimes have been found to cause variations in DOC leaching during the winter season (Fuss et al., 2016). Further research is needed on the role of C dynamics in N retention over the course of the nongrowing season. These seasonal factors represent another facet in understanding the trends in watershed N dynamics.

The evidence presented herein and elsewhere indicates that soil C and N dynamics are responsive to changes in S deposition, but considerable evidence also indicates that C and N dynamics are responsive to variations in temperature and precipitation (Conant et al., 2011; Paul et al., 2003). However, the positive correlation between precipitation and watershed N export during the high retention season in South Buck was the only climate-N relationship observed in this study. Because there was no trend in stream flow in this watershed, the relationship could not be attributed simply to increased soil water flux. Increased precipitation is often positively correlated with increased basal area growth (which was measured in this watershed), but higher growth would be expected to increase N retention, whereas we observed an increase in watershed export in the high retention season. The increase in precipitation may have helped to stimulate decomposition to a level that more than compensated for N assimilated through tree growth, thereby increasing watershed N export. However, in North Buck, C content of the upper soil increased in the Oe and did not change in the Oa, although precipitation levels were likely to be highly similar to those of nearby South Buck. These relationships suggest that trending climate did not play a large role in the changes that occurred in these watersheds during the study period.

### 4.4. Possible Interactions Between Nitrogen and Calcium Availability

The importance of varying watershed characteristics in N retention and release has received considerable research attention, but only limited information is available on interactions between soil-Ca availability and forest N dynamics. The ongoing wollastonite addition experiment in New Hampshire has provided some of the most relevant and recent results on a possible link between Ca availability and watershed N release. This one-time addition to a Ca-depleted watershed had minimal effect on N release for about a decade, but over the ensuing 3 to 4 years, stream ${{\text{NO}}_{3}}^{-}$ concentrations increased abruptly, shifting the watershed from an N sink to an N source (Rosi-Marshall et al., 2016). Further work in this watershed indicated that release of N from soil to stream became more sensitive to hydrologic events during the period of high N release (Marinos et al., 2018). Investigators considered these findings to be consistent with a watershed recovering from prior acidification and N saturation (Marinos et al., 2018).

The wollastonite treatment had the dual effect of neutralizing acidified soils beyond natural recovery and increasing Ca availability for tree growth. However, through the course of the experiment, Ca availability may have declined, while soil became less acidic from the ambient decrease in acidic deposition. These factors could have opposing effects on watershed N release that would complicate interpretation of the recovery response. The wollastonite experiment and the Buck Creek monitoring both suggest increased N release from accelerated decomposition in recovering soils, although the conditions created by the wollastonite addition differed somewhat from the Adirondack conditions where soil acidity decreased gradually and Ca availability was relatively stable. Nevertheless, our regional stream data representing a large number of watersheds with wide variations in soils and acidic deposition effects provide further empirical support for Ca availability as a contributing factor in controlling watershed N retention and release.

Other nutrients, such as phosphorus (P), have been considered as potentially growth limiting in N-saturated forest ecosystems. A recent N-P manipulation experiment in New Hampshire soils with a range in Ca availability similar to the Adirondack region (Goswami et al., 2018) did observe increased growth from P additions in young stands (30–40 years after clearcutting), but not in mature stands, and the only species to significantly respond to treatment was white birch (*Betula papyrifera* Marsh.), a fast-growing, shade-intolerant species that responds strongly to disturbance (Burns & Honkala, 1990). In contrast, the evidence of Ca as growth limiting to key eastern North American species such as sugar maple and red spruce is considerable in Ca-depleted soils (Battles et al., 2014; Bishop et al., 2015; Halman et al., 2013; Hawley et al., 2006; Long et al., 2015) and may include other species such as *Betula alleghaniensis* Britt. (Ouimet et al., 2017) and *Fraxinus americana* L. (Lawrence, McDonnell, et al., 2018).

North and South Buck related well to the regional stream data by bracketing ${{\text{NO}}_{3}}^{-}$ concentrations for most of the streams with low acid buffering (${\text{Ca}}^{2+}$/${{\text{SO}}_{4}}^{2-}$ < about 1.5), as well as highlighting the variability in processes controlling N retention and release. The increases and decreases of ${{\text{NO}}_{3}}^{-}$ measured in the various streams between the 2004–2005 and 2014–2015 surveys also suggested that the variations in N dynamics seen in North and South Buck were likely to occur across the region. These sources of variability in soil and vegetation processes appear to have obscured any effect that may have resulted from the small N deposition decrease of approximately 2 kg·ha^−1^·year^−1^ that occurred in the interval between surveys. Five streams in the Catskill region, NY, also showed no trends in stream ${{\text{NO}}_{3}}^{-}$ concentrations from a decrease in N deposition of approximately 8 kg·ha^−1^·year^−1^ from 1992 to 2014 (McHale et al., 2017). In contrast to findings in NY, nine predominantly forested watersheds in Pennsylvania, Maryland, and Virginia, USA, did exhibit strong decreases in ${{\text{NO}}_{3}}^{-}$ concentrations and watershed export for 1986–2009, which roughly coincided with decreases in N deposition (Eshleman et al., 2013). The cause of the contrasting results is unclear, and direct comparisons are difficult because these mid-Atlantic watersheds were much larger than the Adirondack watersheds.

## 5. Conclusion

Watershed N dynamics have been viewed in terms of N deposition regimes since the development of the N saturation concept (Aber et al., 1998). However, the results of this study identify changes in S deposition rather than N deposition as the key driver in watershed N export during the study period. This process, although seemingly tied to recovery, is causing continued mobilization of toxic levels of Al_i_ in the study region, thereby impeding biological recovery of streams (Baldigo et al., 2019). Sustained high levels of N export can be explained by higher decomposition rates driven by the pronounced decline in S deposition, but the strength of this mechanism varies among watersheds. The possibility has been raised that increased transfer of atmospheric C to the soil could occur with longer growing seasons and higher atmospheric CO_2_ concentrations, which could actually make N less available for plant growth and lead to N oligotrophication in northern hardwood forests (Groffman et al., 2018) and elsewhere (Craine et al., 2018). However, this has not yet happened in the Adirondack region, and in addition to the results of McHale et al. (2017), similar releases of N have been recently documented in the Czech Republic (Oulehle et al., 2011) and Germany (Meesenburg et al., 2016). The range over which S deposition decreased in the European studies was much greater than in this study, which suggests that the effect of decreasing S deposition may be applicable not only to regions that received the highest deposition levels but also to those that received more moderate levels in the past.

Without improved Ca availability, limited acid buffering will mean that ${{\text{NO}}_{3}}^{-}$-driven acidification episodes dependent on seasonal shifts in plant-microbe interactions and longer-term trends in soil C availability will likely continue. At this stage in the recovery process, stream water impacts from watershed N release appear to be related to the effects of decreased S on C dynamics, although the cumulative effect of N deposition over the past decades was likely to have contributed to a condition of N saturation. The elevated watershed release of N over the 16-year study period may only represent a phase of recovery, and with continued low levels of N deposition and increases in forest productivity, these watersheds may resume high levels of N retention and associated water quality benefits (Gilliam et al., 2019). However, considerable uncertainty remains regarding the future capacity of watersheds to retain N and the rate at which retention can occur. In the present, streams with harmful Al concentrations linked to ${{\text{NO}}_{3}}^{-}$ release remain common (Lawrence, Burns, & Riva-Murray, 2016).

The remaining question of how long watershed release of N will continue in these types of watersheds will likely be tied to whether the changes in C dynamics and Ca availability observed in this study will continue. Because organic C dynamics are sensitive to precipitation and temperature, the effect of climate trends on N retention and release may increase in the future.

## Supplementary Material

Supplement1

## Figures and Tables

**Table 1:** Mean Values of Basal Area, Tree Biomass (above and belowground), Foliage Biomass, and Wood N and Foliage N of Live Trees >5-cm diameter at breast height

Tree Measurement	Year			
	2000	2005	2010	2015
Basal area (m^2^/ha)				
North Buck	31.1 (21)	31.3 (24)	29.7 (25)	31.4 (26)
South Buck*	28.8 (29)	30.9 (30)	32.8 (30)	33.5 (28)
Tree biomass (Mg/ha)				
North Buck	207 (20)	213 (22)	209 (23)	222 (23
South Buck*	253 (39)	272 (39)	289 (39)	293 (34)
Foliage biomass (Mg/ha)				
North Buck	10.1 (43)	10.3 (48)	9.2 (54)	9.8 (55)
South Buck*	5.2 (27)	5.5 (27)	5.9 (27)	6.1 (23)
Wood N (kg N/ha)				
North Buck	180 (26)	185 (23)	185 (26)	195 (26)
South Buck*	263(40)	283 (39)	300 (39)	304 (33)
Foliage N (kg/ha)				
North Buck	140 (31)	142 (32)	131 (34)	138 (36)
South Buck*	109 (28)	116 (28)	122 (29)	125 (26)

*Note*. Coefficient of variation for the 15 plots in each watershed is given in parentheses.

* Significant trend (*P* < 0.05) over the 15 years.

**Table 2:** Mean Soil Data for Initial and Final Samplings of North and South Buck Watersheds for Oe, Oa, and Upper 10 cm of the B Horizon

	North Buck				South Buck			
		
Soil Property	1997	2009–2010	*P*	95% CL	1998	2014	*P*	95% CL
Oe Horizon								
C(g/kg)	460	513	**	503–523	427	421	**	
C (Mg/ha)	29	32			23	16		12–20.6
CEC (cmol_c_/kg)	26	32	**	29–33	24	22	**	73–93
Base sat. (%)	60	65			50	85		
pH (0.01 CaCl_2_)	3.02	3.16	**	3.07–3.24	3.35	3.36		
H (cmol_c_/kg)	8.4	9.9	*	8.5–11.2	5.6	2.4	**	0.5–4.0
Al sat. (%)	9.7	2.6	**	0–5.8	27	3.4	**	0–14
Al:C (g:g)	0.0005	0.0001	**	0–0.0003	0.0014	0.0002	**	0–0.0008
N (g/kg^1^)	22	25	**	23–26	22	23	**	0.68–1.1
N (Mg/ha)	1.4	1.5			1.2	0.89		
C:N (g:g)	21	21			20	18		
Oa Horizon								
C(g/kg)	416	466	**	432–499	378	366	**	11.9–19.1
C Mg (ha)	83	91			24	15		
CEC (cmol_c_/kg)	31	31			20	15		
Base sat. (%)	30	31			43	49		
pH (0.01 CaCl_2_)	2.69	2.74			3.15	3.17		
H (cmol_c_/kg)	11	13			5.2	4.5		
Al sat. (%)	35	26	*	34–18	29	21	**	0.0002–0.0013
Al:C (g:g)	0.0023	0.0016	*	0.0022–0.0010	0.0016	0.0008		
N(g/kg)	17	20	**	19–22	18	18		
N (Mg/ha)	3.2	3.7	*	3.3–4.2	1.2	0.74		
C:N (g:g)	25	23	*	24–22.2	20	20		
Upper B Horizon								
C(g/kg)	80	91			57	60		
C (Mg/ha)	35	35			27	20	**	17–22
CEC (cmol_c_/kg)	5.2	11	**	9.3–12	3.4	4.6	**	4.0–5.3
Base sat. (%)	12	6.1	**	4.3–7.9	15	9.6	**	5.7–13
pH (0.01 CaCl_2_)	3.35	3.53	**	3.42–3.63	3.79	3.74		
H (cmol_c_/kg)	1.4	1.4			0.51	0.42		
Al sat. (%)	64	81	**	81–87	71	85	**	76–93
Al:C (g:g)	0.0044	0.0085	**	0.0082–0.0097	0.0040	0.0061	**	0.0051–0.0071
N(g/kg)	3.4	3.6			2.7	2.6		
N (Mg/ha)	1.5	1.3			1.3	0.85	**	0.72–0.98
C:N (g:g)	26	26			21	24	**	21.4–25.9

*Note*. Differences between watersheds for each sampling period are indicated by pink (*P* < 0.05) and red (*P* < 0.01) shading of South Buck columns. Differences between samplings within watersheds are indicated by * (*P* < 0.05) and ** (*P* < 0.01). The 95% CL (confidence limits) present the limits of the change (defined by *P* < 0.05) in relation to the original measurement in 1997 or 1998 for all significant changes (*P* < 0.05).

**Table 3:** Two Pathways That Encompass Watershed Responses Across the Adirondack Region to High Levels of Acidic Deposition Followed by Large Decreases in Acidic Deposition

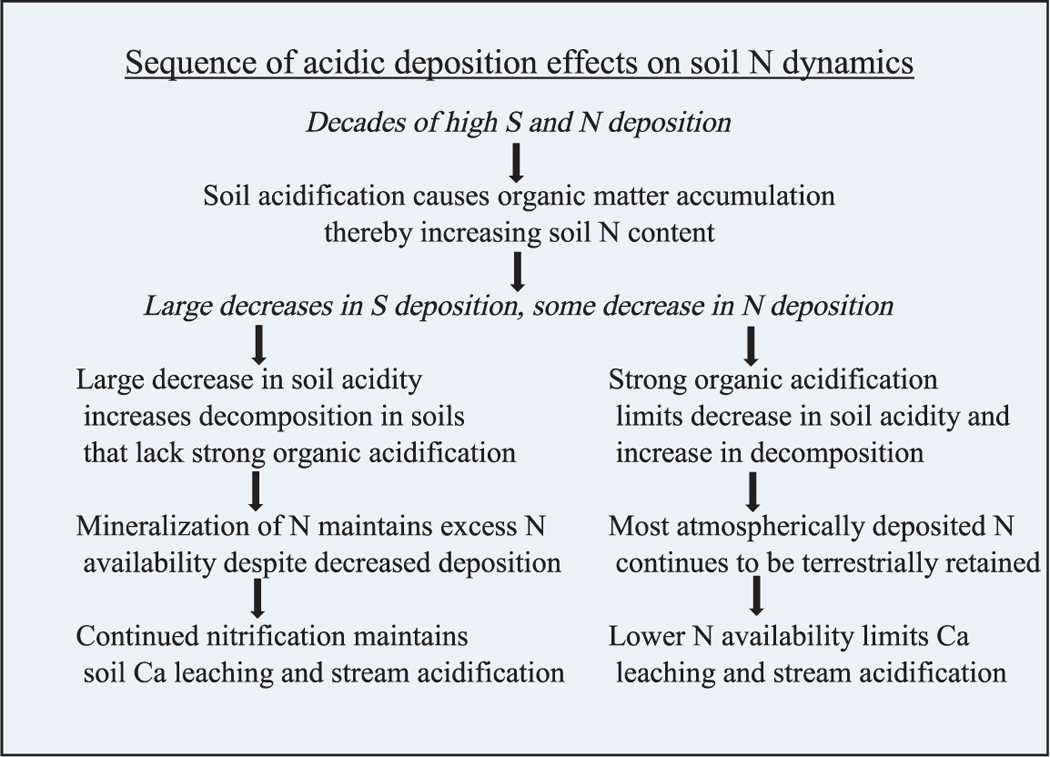

*Note*. Italicized text indicates the change in acidic deposition; non-italicized text indicates the chain of subsequent effects on soil and stream water.

## Key Points:

- Decreasing atmospheric deposition of nitrogen has not lowered watershed export of nitrogen by Adirondack streams
- Long-term decreases in acidifying sulfur deposition are stimulating decomposition and nitrogen mineralization in the forest floor
- Movement of harmful nitric acid and aluminum from soils to streams continues despite lower deposition of sulfur and nitrogen
