# Supplementary material for: Recovery of Soils From Acidic Deposition May Exacerbate Nitrogen Export From Forested Watersheds
Source: J Geophys Res Biogeosci. Author manuscript; Available in PMC 2024 Oct 7. (PMC11457161; doi:10.1029/2019jg005036)
Supplement: Supplement1 [file NIHMS2002159-supplement-Supplement1.docx]

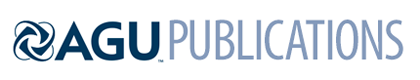


Journal of Geophysical Research Biogeosciences Supporting Information for

Title: Recovery of soils from acidic deposition may exacerbate nitrogen export from forested watersheds

Gregory B. Lawrence^1^, Sara E. Scanga^2^, Robert D. Sabo^3^

^1^ U.S. Geological Survey, New York Water Science Center, Troy, NY, 12180; [glawrenc@usgs.gov](mailto:glawrenc@usgs.gov),

^2^Department of Biology, Utica College, 1600 Burrstone Rd, Utica, NY 13037, USA, [sescanga@utica.edu](mailto:sescanga@utica.edu)

^3^Oak Ridge Institute for Science and Education Research Participant, United States Environmental Protection Agency, Office of Research and Development, US EPA (8623-P);1200 Pennsylvania Ave NW; Washington, DC 20460, [sabo.robert@epa.gov](mailto:sabo.robert@epa.gov)

Corresponding author: Gregory B. Lawrence^1^ ([[glawrenc@usgs.gov](mailto:glawrenc@usgs.gov))](mailto:email@address.edu))

**Contents of this file**

Text S1 to S4

Figures S1 to S3

Tables S1 to S3

**Introduction**

Supporting Information includes additional details on methods pertaining to the chemical analysis of North and South Buck Creek stream water samples (S1), the sampling design and chemical analysis of stream samples collected in the Western Adirondack Stream Survey (WASS) and the East-Central Adirondack Stream Survey (ECASS) (S2), soil sampling and chemical analysis of collected soils, and methods for estimating biomass (S3) and N content of trees in the North and South Buck Creek watersheds (S4). This information is supported by Figures S1-S3 and Tables S2-S3. Table S1 lists the location coordinates and matches the NWIS database codes with project stream codes to aid in downloading stream flow and chemistry data from the USGS National Water Information System (NWIS) database.

S1 North and South Buck Creek Chemical Analysis of Water Samples

All North and South Buck stream samples collected from January 2000 through September 2008 were analyzed for NO_3_^-^, NH_4_^+^, SO_4_^2-^ Ca^2+^, and dissolved organic carbon (DOC). All stream analyses were done at the USGS New York Water Science Center Soil and Low-Ionic Strength Water Quality Laboratory in Troy, New York (NY), USA (hereafter the USGS Troy laboratory). Stream samples collected from October 2008 through December 2015, were analyzed for these same constituents at the laboratory of the Adirondack Lake Survey Cooperation (ALSC), Ray Brook, NY. In both laboratories, the same U.S. Environmental Protection Agency (USEPA) methods were used for these analyses ([https://nepis.epa.gov/Exe/ZyPDF.cgi?Dockey=30000TA0.PDF](https://www.nrcs.usda.gov/wps/portal/nrcs/detail/soils/ref/?Dockey=30000TA0.PDF); accessed January 15, 2018).

Samples collected in these watersheds from October 1999 through September 2008 were also analyzed for total dissolved nitrogen (TDN) at the USGS Troy laboratory using colorimetric detection after persulfate/ultraviolet digestion and cadmium reduction with an automated flow-injection analyzer. All stream chemistry data for these sampling locations are available from the USGS National Water Information System (NWIS) database (U.S. Geological Survey, National Water Information System—Web interface, accessed at http://dx.doi.org/10.5066/F7P55KJN, March 28, 2019). NWIS database codes and coordinates for the stream sampling locations are listed in Table S1.

To compare results obtained by the USGS Troy laboratory and the ALSC laboratory, duplicate samples were collected from North and South Buck Creek and the main stem of Buck Creek biweekly from September 5, 2006, to September 2, 2008. Each laboratory received one of the duplicates for a total of 53 samples from main stem Buck and South Buck, and 49 samples from North Buck, which was dry at the sampling location on four of the sampling dates. The 155 samples collected over 2 years enabled comparisons to be made under a variety of flows and seasons, with the differing chemistries of the three streams. Results yielded mean concentration differences between laboratories of 0.5 mmol L^-1^ for NO_3_^-^, 0.8 mmol L^-1^ for NH_4_^+^, 0.7 mmol L^-1^ for SO_4_^2-^, and 4.9 mmol L^-1^ for Ca^2+^. Linear regression performed on results of the two laboratories indicated strong linear relationships (Figure S2), with slopes ranging from 0.762 to 0.943, and 16% or less unexplained variability, except for NH_4_^+^. Nearly all NH_4_^+^ concentrations were less than 1 mmol L^-1^ and most were less than 0.5 mmol L^-1^, which is typical for these streams. Because most of these concentrations approached the limit of detection, precision and accuracy of the measurements were low, which resulted in the weak correlation.

Concentrations of dissolved organic nitrogen (DON) were obtained by subtracting concentrations of NO_3_^-^ and NH_4_^+^ from TDN. Because TDN analysis was not run on stream samples through the full study period, DOC was used to estimate DON to complete the record. Simple linear regression was applied to samples with both measurements to the develop the following relationships between DON and DOC.

North Buck DON = -6.747 + (0.0213 * North Buck DOC); P < 0.01, R^2^ = 0.18, n = 257

South Buck DON = -4.405 + (0.0417 * South Buck DOC); P < 0.01, R^2^ = 0.12, n = 254

For the period that TDN was not measured, mean monthly values of DON were added to those of NO_3_^-^ and NH_4_^+^ to obtain mean monthly values of TDN concentrations that could be combined with flow data to estimate TDN export from each watershed for the entire record.

**S2 WASS and ECASS Regional Sampling Design and Chemical Analysis**

In the Western Adirondack Stream Survey (WASS) and East-Central Adirondack Stream Survey (ECASS) studies, streams were identified for possible sampling if they met the following criteria: (1) the stream appeared on a USGS topographic map coverage at 1:24,000 scale, (2) the stream was estimated to be accessible by hiking to and from the sampling location within about 1 hour, and (3) the stream did not contain upstream lakes, ponds or wetlands that drained more than 25% of the total drainage area defined by the sampling point. As a result of these criteria, all of the streams identified for potential sampling could be considered low-order (mostly first order) headwater streams. From this set of streams, a random selection procedure was used to identify 200 streams for sampling both in the WASS and ECASS regions (Figure S1). Sampling dates were designed to account for temporal variations in chemistry that vary with flow and season. Flow-driven variations in chemistry that can occur hourly or daily are problematic for regional surveys such as this because flow and stream chemistry can vary over the length of time needed to reach all the sites. To reduce this variability, the sampling periods of each survey were selected when flows were expected to remain either elevated (primary spring snowmelt) or low (August base flow) for several days. In nearly all surveys, sampling was completed within 3 consecutive days.

For WASS and ECASS surveys, all samples were analyzed for NO_3_^-^, Ca^2+^ and SO_4_^2-^ in the ALSC laboratory with USEPA methods ([https://nepis.epa.gov/Exe/ZyPDF.cgi?Dockey=30000TA0.PDF](http://waterdata.usgs.gov/nwis/?Dockey=30000TA0.PDF); accessed January 15, 2018). All stream chemistry data for these sampling locations are available from the USGS NWIS database (U.S. Geological Survey, National Water Information System—Web interface, accessed at http://dx.doi.org/10.5066/F7P55KJN, March 28, 2019). NWIS database codes and coordinates of the stream sampling locations are listed in Table S1.

**S3 Soil Sampling and Analyses**

Determining whether the horizon underlying the Oe was Oa or A in the field was difficult in some soil profiles in South Buck. Laboratory analysis identified C concentrations less than 20% in two samples identified in the field as Oa in the 1998 sampling and five samples identified in the field as Oa in the 2014 sampling. Because these samples did not meet the Oa requirement of at least 20% C (Schoeneberger et al., 2012), they were excluded from this analysis, which resulted in n = 26 for 1998 Oa samples, and n = 23 for 2014 Oa samples in South Buck.

Organic samples were ground through a 1.0-mm sieve and mineral samples were passed through a 2-mm sieve. Samples were analyzed for moisture content (oven drying at 65 and 105°C for organic and mineral samples, respectively), exchangeable Ca, Mg, Na, and K (unbuffered 1 M NH_4_Cl vacuum extraction), loss-on-ignition, and pH (0.01 M CaCl_2_ slurry) following USEPA methods (Blume et al., 1990). Exchangeable Al was determined by 1 M KCl batch extraction or 1 M NH_4_ vacuum extraction and measurement by inductively coupled plasma optical spectrometer (ICP). Concentrations of C and N were determined by a thermo-combustion elemental analyzer using the methods of the instrument manufacturer. Exchangeable acidity was determined by 1 M KCl batch extraction and measurement by titration (Thomas, 1982). Exchangeable H was calculated by subtracting exchangeable Al from exchangeable acidity. Effective cation exchange capacity (CEC) was calculated as the sum of the exchangeable forms of acidity, calcium (Ca), magnesium (Mg), potassium (K), and sodium (Na). Base saturation was calculated as the sum of exchangeable base cations (Ca, Mg, K, and Na) divided by the CEC.

Unused portions of soil samples collected in 1997 (North Buck) and 1998 (South Buck) were archived, and 27 samples (9 per horizon) were reanalyzed with the samples collected in 2014. Results were compared to evaluate possible analytical biases between analyses of the different collections and to adjust data as shown in Supplemental Methods of Lawrence et al. (2018). All soil data are available as a USGS data release (Lawrence et al., 2017).

**S4 Methods for Estimating Biomass and N Content of Trees**

For the complete tree biomass estimates, species-specific allometric equations were available from Young et al. (1980), summarized in Jenkins et al. (2004) for all species except black cherry (*Prunus serotina* Ehrh.) and striped maple (*Acer pennsylvanica* L.); we substituted the pin cherry (*Prunus pensylvanica* L. f.) and red maple (*Acer rubrum* L.) equations from Jenkins et al. (2004) for these two species, respectively. For the foliage biomass estimates, the American beech (*Fagus grandifolia* Ehrh.) equation was substituted for black cherry, and the red maple equation was substituted for striped maple. These substitutions likely had only a minor effect on our final estimates for biomass and N storage, because out of 922 different trees sampled in total (North Buck: n = 464; South Buck: n = 458), only 1 was a black cherry (in the North) and 43 were striped maples (North Buck: n = 21 trees; South Buck: n = 22 trees).

Species-specific trunk wood N concentrations were taken from Table 1 in Pardo et al. (2005), who provide mean species-specific bole N concentrations for the Northeast USA for all species found in North and South Buck except black cherry and striped maple. For the striped maple trees, we used the red maple N concentrations from Table 1 in Pardo et al. (2005). For the single black cherry tree, we used an estimate of 0.12% N obtained from a Massachusetts site (Adams et al., 1995) in the Tree Chemistry Database version 1; Pardo et al. (2005). The mean bole N concentrations in Table 1 of Pardo et al. (2005) were similar to bole N concentrations (~1985 – 2009) observed in cores taken from 20 trees of multiple species from each of the watersheds (North Buck mean = 0.10% N; South Buck mean = 0.12% N) in a previous study (Sabo et al., 2016).

Foliage N concentrations were also taken from Table 1 of Pardo et al. (2005), again with the exception of black cherry and striped maple. For these species, foliar N concentration values (black cherry = 3.271%; striped maple = 2.499%) were obtained from the mean of all individual trees sampled in NY (NERC, 2010). Species-specific foliar N concentrations were multiplied by the foliar biomass of each tree to estimate the foliar N storage by individual trees. Complete tree N storage was estimated by summing the foliar and wood N storage estimates for each tree. Next, individual tree foliage, wood, and complete tree N storage estimates were summed for each plot then averaged over all plots. These mean values were extrapolated to the whole watershed.

**References (these also appear in article reference list)**

Adams, M. B., Kochenderfer, J. N., Angradi, T. R., and Edwards, P. J. (1995). Nutrient budgets of two watersheds on the Fernow Experimental Forest, In: Gottschalk, K.W., Fosbroke, S.L.C. ed. Proceedings, 10th Central Hardwood Conference; March 1995, Morgantown W.V.: U.S.D.A. Forest Service General Tech. Rep. NE-197, pp 119-130. <https://www.fs.usda.gov/treesearch/pubs/12745>

Blume, L. J., Schumacher, B. A., Schaffer, P. W., Cappo, K. A., Papp, M. L., van Remortal, R. D., Coffey, D. S., Johnson, M. G., and Chaloud, D. J. (1990). Handbook of methods for acid deposition studies: Laboratory analyses for soil chemistry. U.S. Environmental Protection Agency, EPA/600/4-90/023. <http://nepis.epa.gov/Exe/ZyPDF.cgi?Dockey=200096FB.PDF>.  *EPA/600/4-90/023*, U.S. Environmental Protection Agency, EPA/600/4-90/023.

Jenkins, J. C., Chojnacky, D. C., Heath, L. S., and Birdsey, R. A. (2004). Comprehensive batabase of diameter-based biomass regressions for north american tree species, Gen. Tech. Rep. NE-319. Newtown Square, PA: U.S. Department of Agriculture, Forest Service, Northeastern Research Station. 45 p. <https://www.fs.usda.gov/treesearch/pubs/7058>.

Lawrence, G. B., Sullivan, T. J., Bailey, S. W., McDonnell, J. J., and Antidormi, M. R. (2017). Adirondack New York soil chemistry data, 1997-2014: U.S. Geological Survey data release; <https://doi.org/10.5066/F78050TR> edited.

Lawrence, G. B., McDonnell, T. C., Sullivan, T. J., Dovciak, M., Bailey, S. W., Antidormi, M. R., and Zarfos, M. R. (2018). Soil base saturation combines with beech bark disease to influence composition and structure of sugar maple-beech forests in an acid-rain impacted region. *Ecosystems*, *21*, 1432-9840. <https://doi.org/10.1007/s10021-017-0186-0>

NERC (2010). Compilation of foliar chemistry data for the northeastern United States and southeastern Canada. NERC.12.6. <http://www.nercscience.org>, edited.

Pardo, L. H., Robin-Abbott, M. J., Duarte, N., and Miller, E. K. (2005). Tree chemistry database (version 1.0). Gen. Tech. Rep. NE-324. Newtown Square PA: U.S. Department of Agriculture, Forest Service, Northeastern Research Station. 45 p. <https://www.fs.usda.gov/treesearch/pubs/9464>.

Sabo, R. D., Scanga, S. E., Lawrence, G. B., Nelson, D. M., Eshleman, K. N., Zabala, G. A., Alinea, A. A., and Schirmer, C. D. (2016). Watershed-scale changes in terrestrial nitrogen cycling during a period of decreased atmospheric nitrate and sulfur deposition. *Atmospheric Environment*, *146*, 271-279. <http://dx.doi.org/10.1016/j.atmosenv.2016.08.055>

Schoeneberger, P. J., Wysocki, D. A., Benham, E. C., and Staff, S. S. (2012). Field book for describing and sampling soils, Version 3.0. Natural Resources Conservation Service, National Soil Survey Center, Lincoln, NE. <https://www.nrcs.usda.gov/wps/portal/nrcs/detail/soils/ref/?cid=nrcs142p2_054184>.

Thomas, G. W. (1982). Exchangeable cations. In Methods of Soil Analysis, part 2. in *Agronomy No. 9*. edited by A. L. Page, pp. 159-166, ASA, Madison, WI. <https://dl.sciencesocieties.org/publications/books/tocs/agronomymonogra/methodsofsoilan2>

Young, H. E., Ribe, J. H., and Wainwright, K. (1980). Weight tables for tree and shrub species in Maine. Life Sciences & Agriculture Experiment Station Miscellaneous Report 230; <https://digitalcommons.library.umaine.edu/aes_miscreports/19/>

*
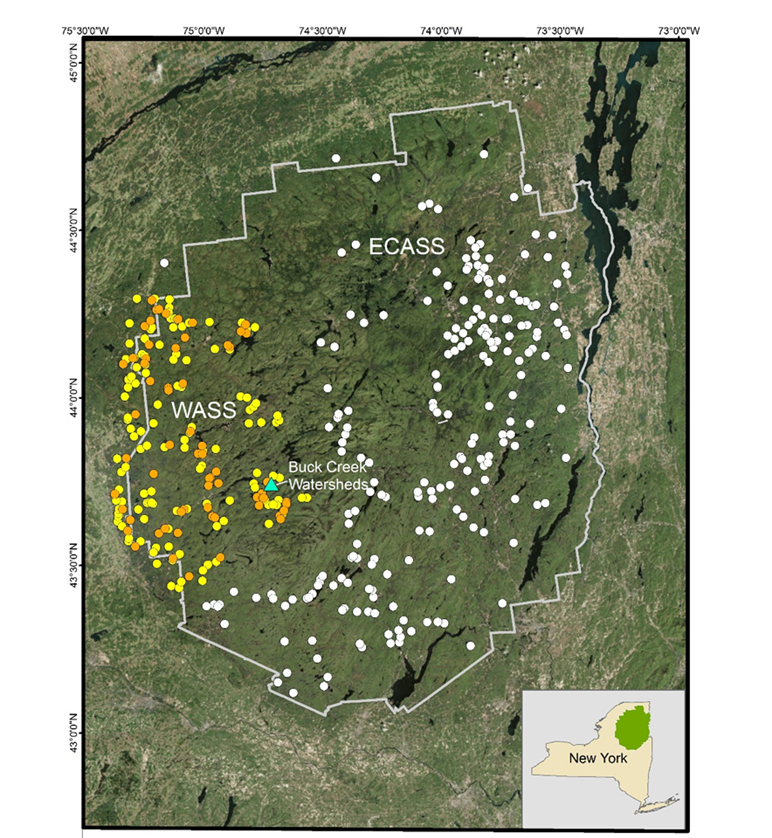
*

**Figure S1.** Stream sampling locations in the 2003/05 Western Adirondack Stream Survey (WASS) shown in yellow; WASS streams resampled in 2014/15 shown in orange. Locations of streams sampled in the 2010/11 East-Central Adirondack Stream Survey (ECASS) shown in white. Location of the North and South Buck Creek watersheds is indicated by the green triangle; gray border indicates the administrative boundary of the New York State Adirondack Park Agency.

**Figure S2**. Results of analyses of stream chemistry done in the USGS (U.S. Geological Survey) Troy Laboratory plotted versus results from analyzing duplicate samples in the ALSC (Adirondack Lake Survey Corporation) laboratory. The equation of the best-fit line and the coefficient of determination are shown for each analysis.

**Figure S3**. Watershed N export in stream water from North and South Buck watersheds for the low N retention season (Dec., Jan. Feb., Mar., and April), and the high N retention season (Jun., Jul., Aug., Sep., and Oct.). P > 0.10 indicates no trend with time.


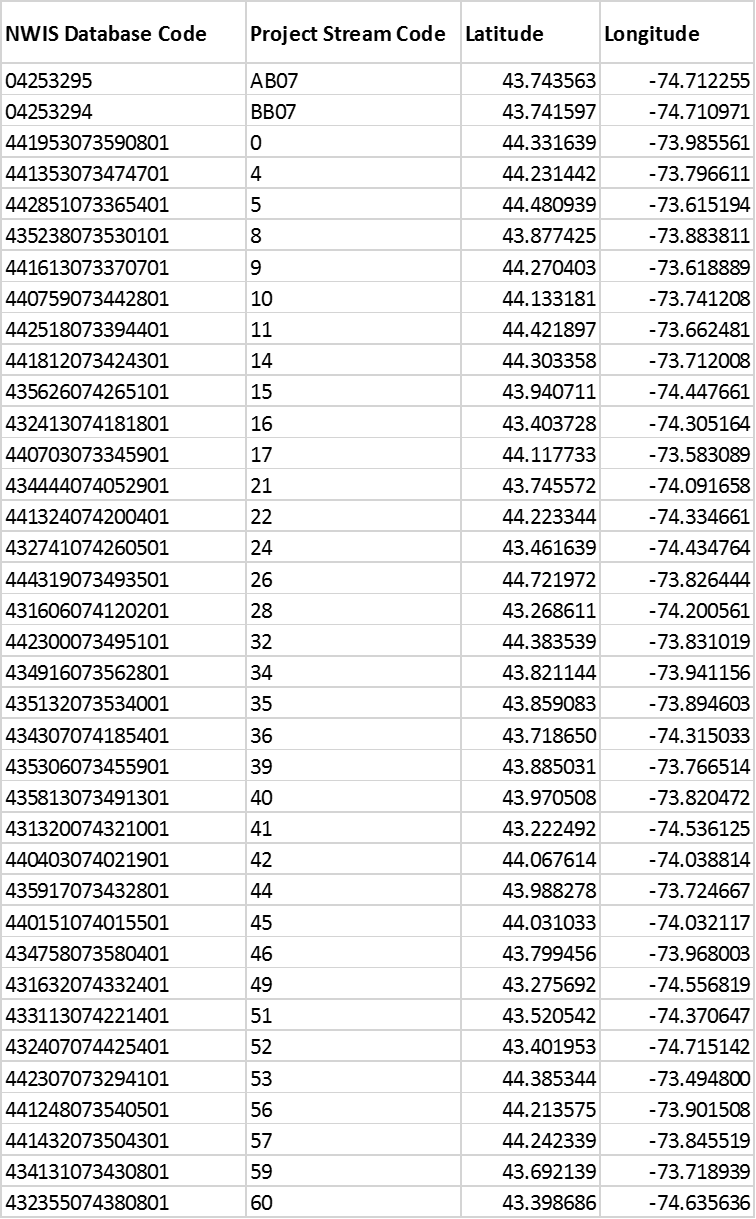


**Table S1-a**. NWIS database codes, project stream codes and coordinates for all streams sampled in the study.


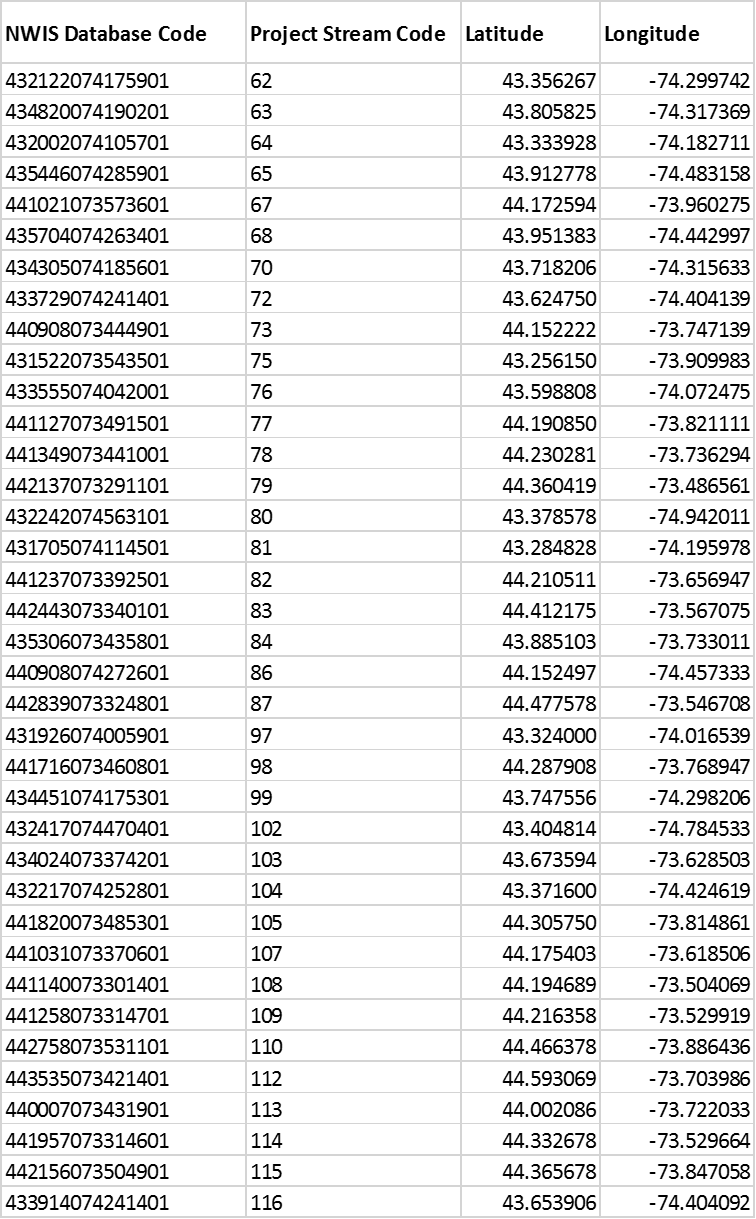


**Table S1-b**. NWIS database codes, project stream codes and coordinates for all streams sampled in the study


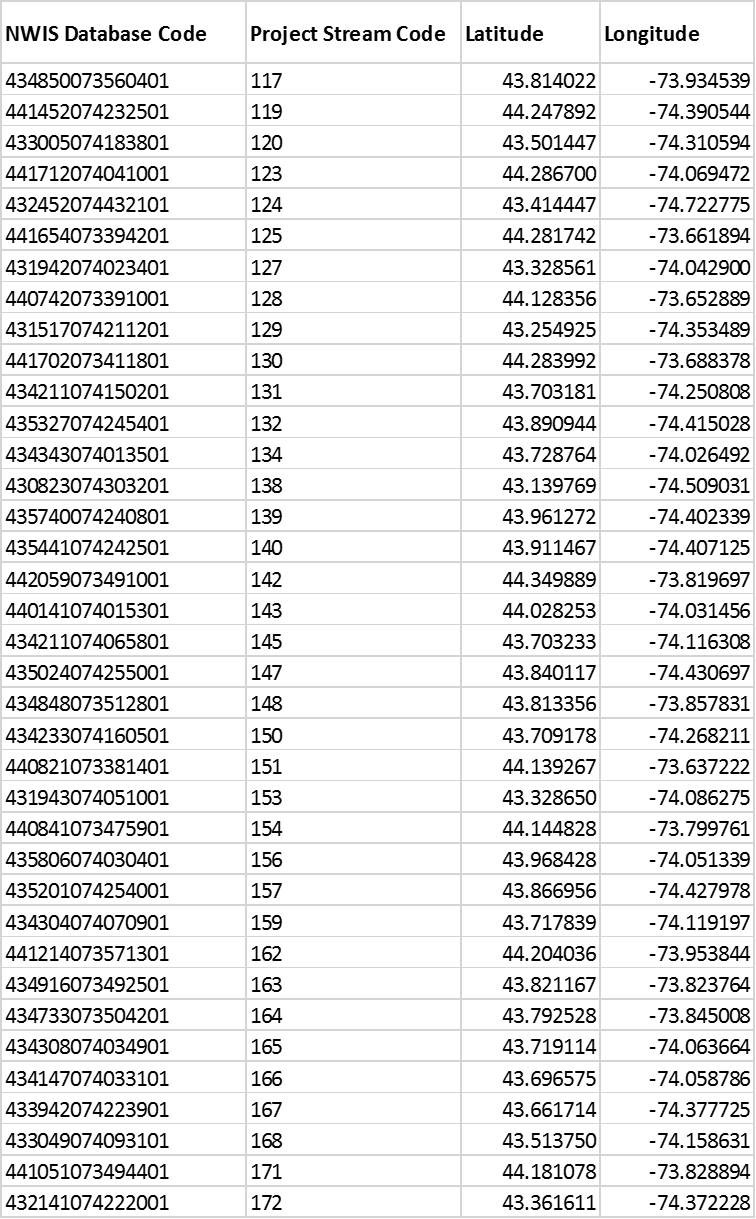


**Table S1-c**. NWIS database codes, project stream codes and coordinates for all streams sampled in the study.


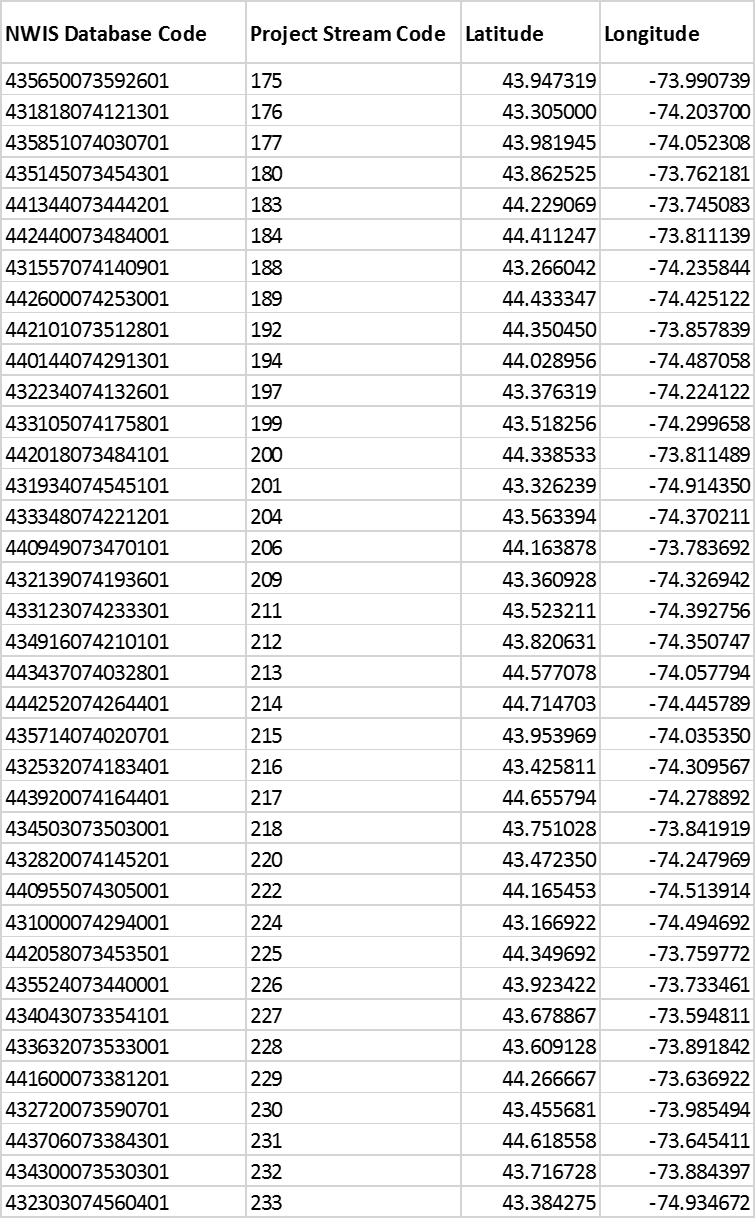


**Table S1-d**. NWIS database codes, project stream codes and coordinates for all streams sampled in the study.


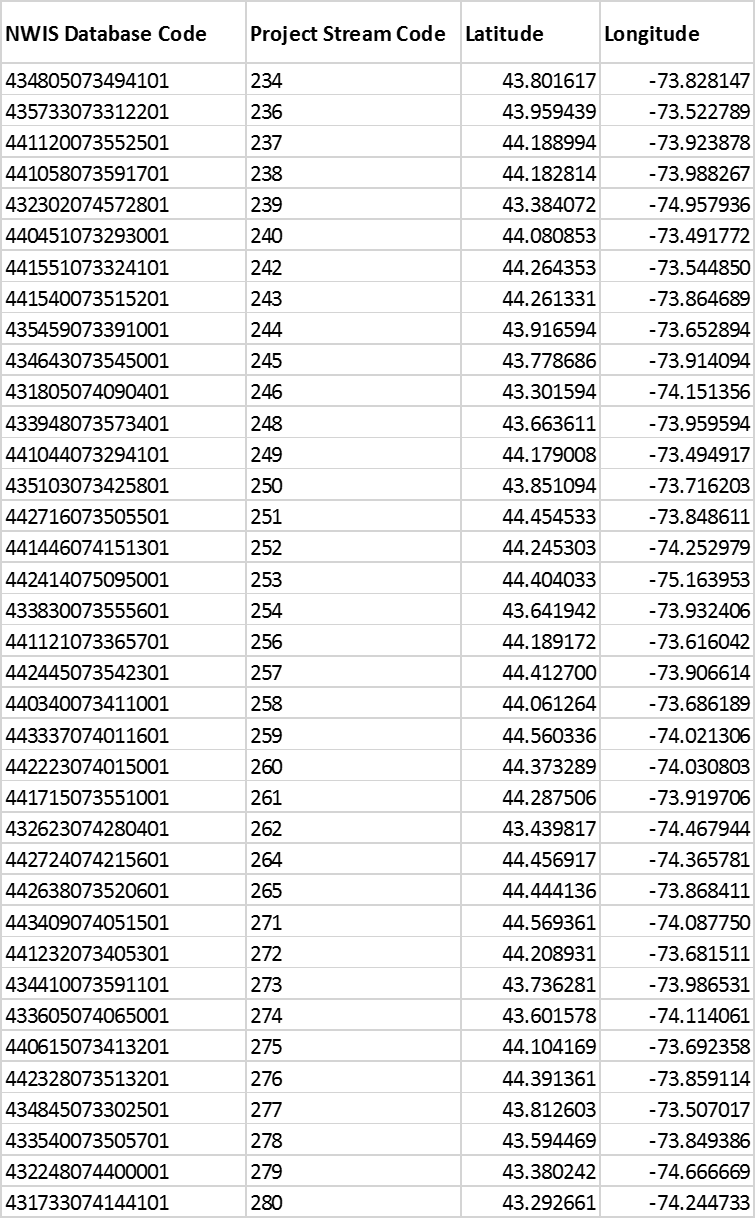


**Table S1-e**. NWIS database codes, project stream codes and coordinates for all streams sampled in the study.


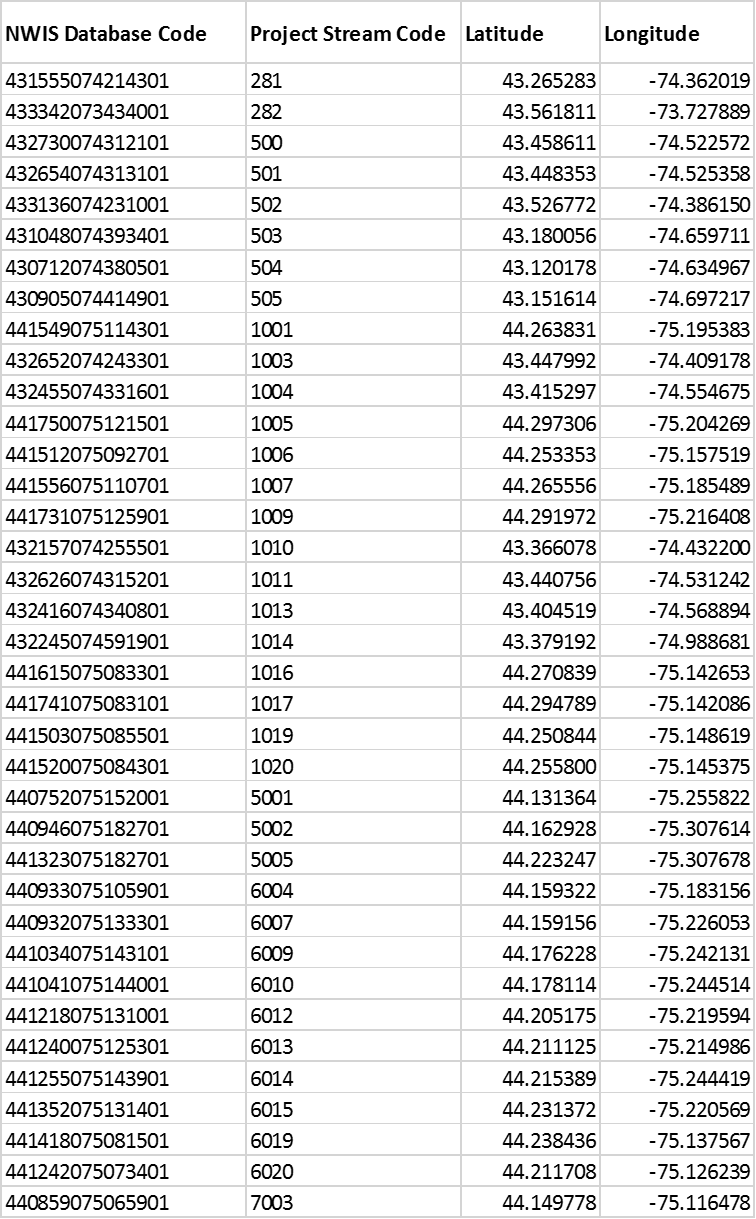


**Table S1-f.** NWIS database codes, project stream codes and coordinates for all streams sampled in the study.


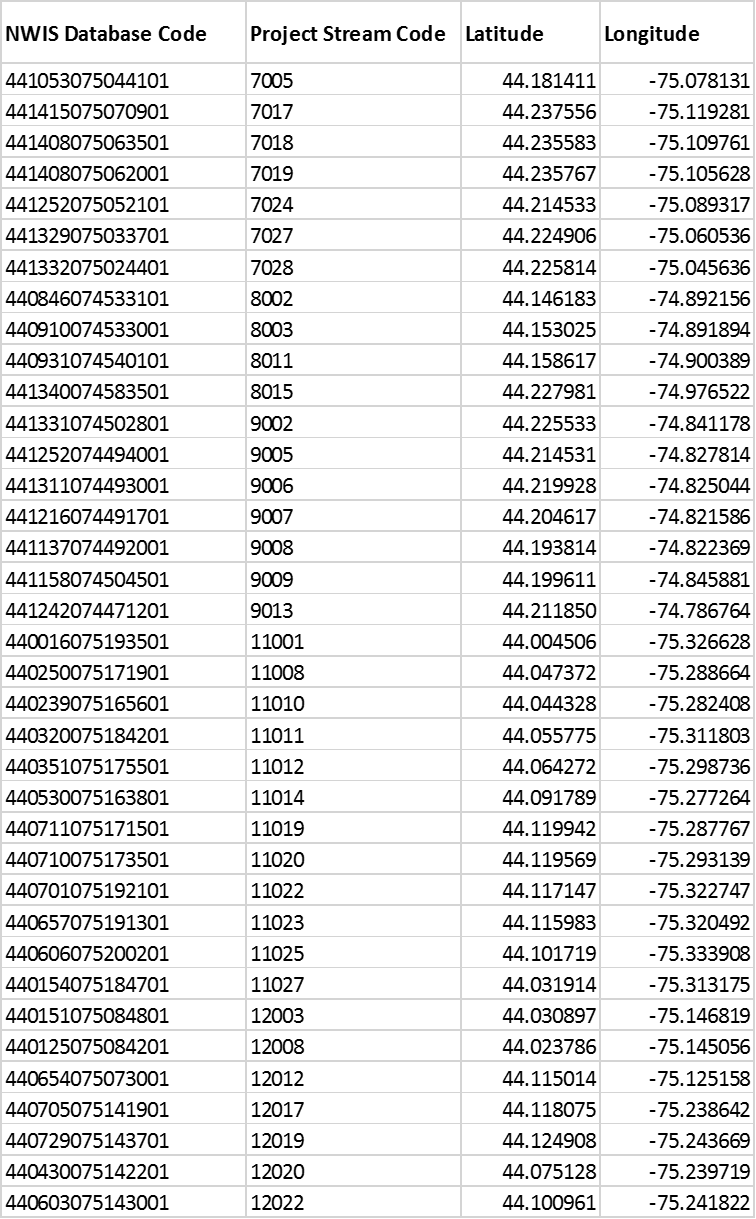


**Table S1-g**. NWIS database codes, project stream codes and coordinates for all streams sampled in the study.


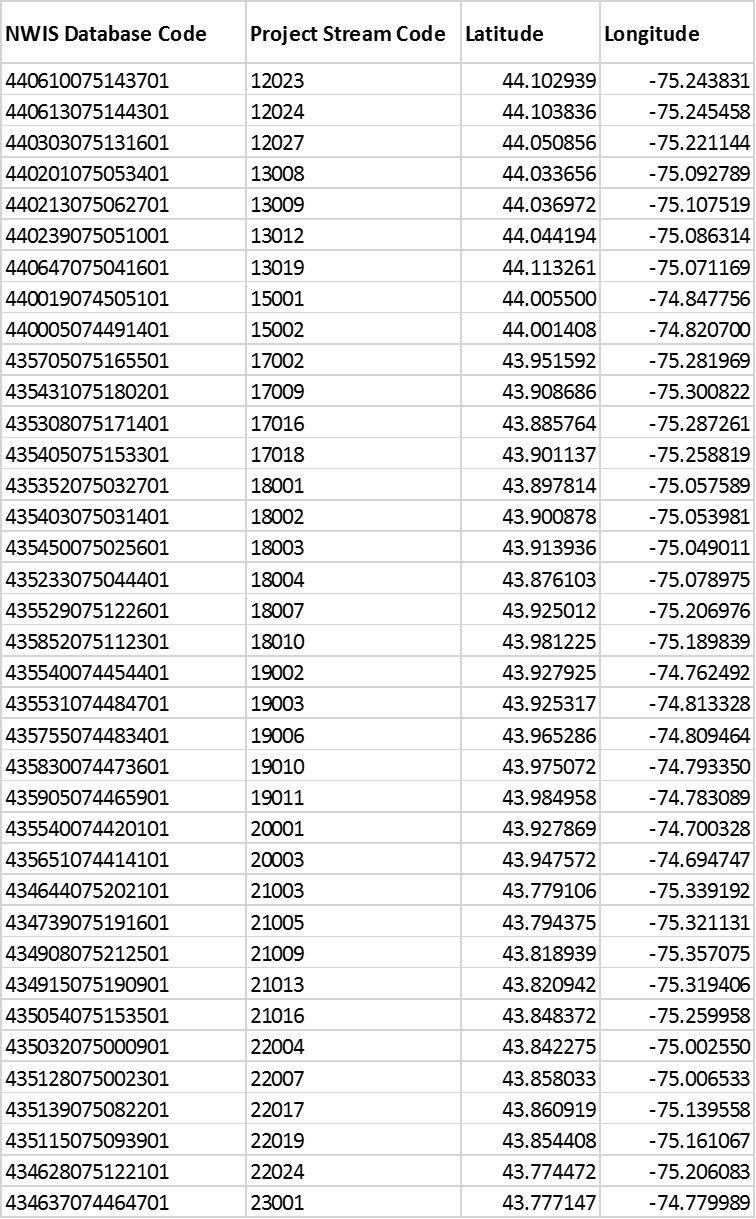


**Table S1-h**. NWIS database codes, project stream codes and coordinates for all streams sampled in the study.


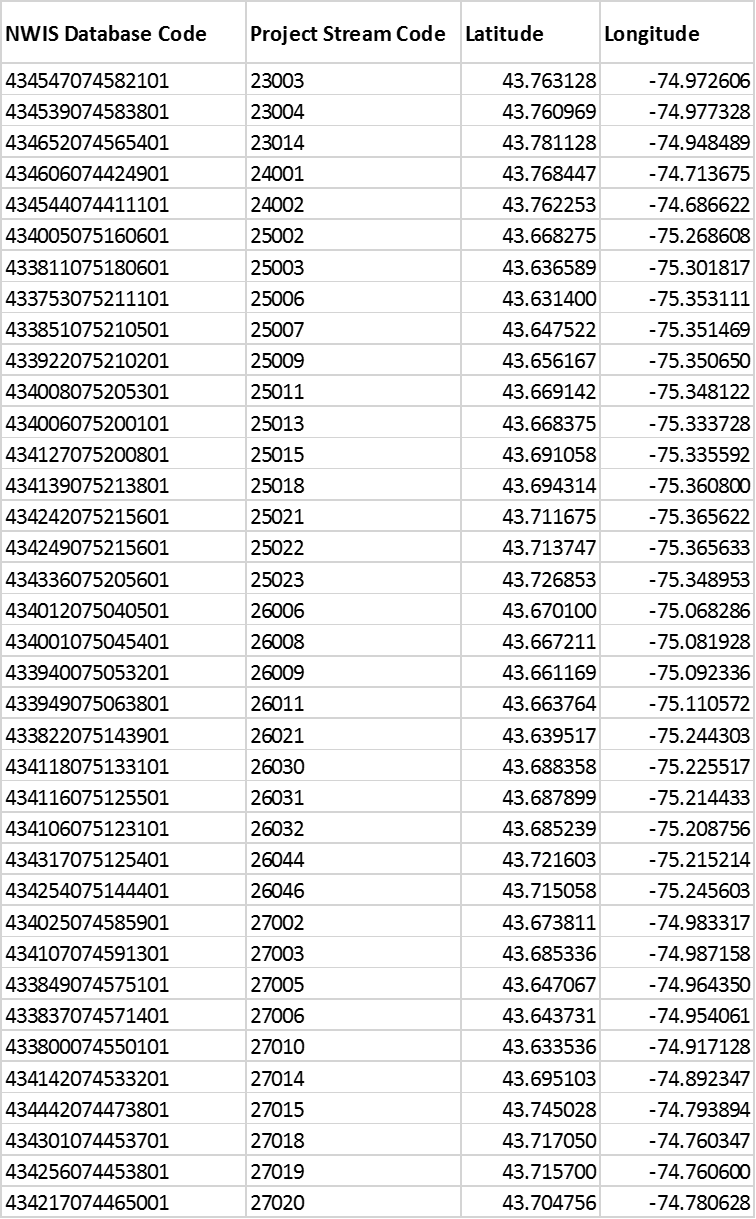


**Table S1-i**. NWIS database codes, project stream codes and coordinates for all streams sampled in the study.


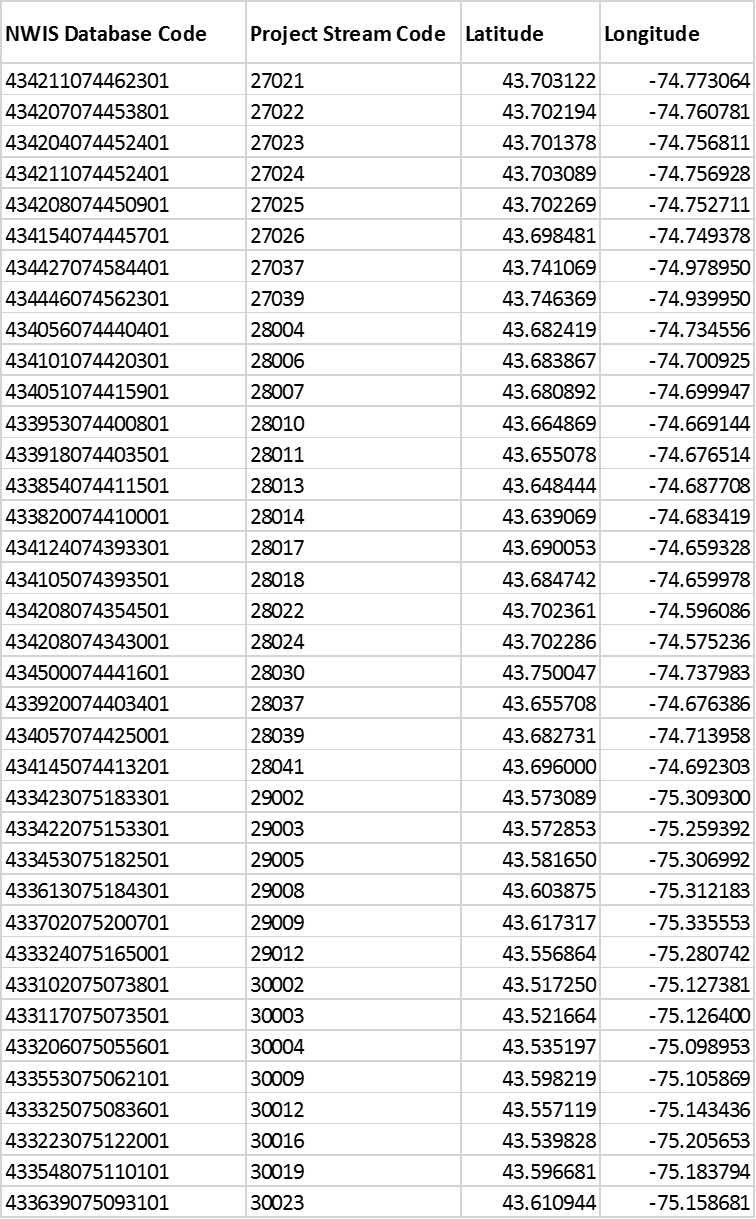


**Table S1-j.** NWIS database codes, project stream codes and coordinates for all streams sampled in the study.


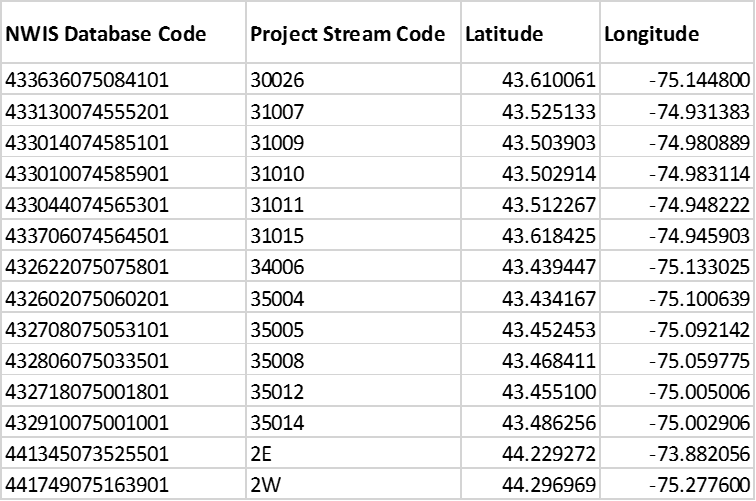


**Table S1-k.** NWIS database codes, project stream codes and coordinates for all streams sampled in the study.

|  | **North Buck** | | **South Buck** | |
| --- | --- | --- | --- | --- |
| **Oe Horizon** | **1997** | **2009-10** | **1998** | **2014** |
|  | **SD** | **SD** | **SD** | **SD** |
| C (g kg^-1^) | 23 | 15 | 61 | 56 |
| C (Mg ha^-1^) | 5.1 | 7.6 | 8.9 | 5.5 |
| CEC (cmol_c_ kg^-1^) | 4.3 | 4.9 | 9.0 | 4.9 |
| Base sat. (%) | 12 | 13 | 21 | 8.1 |
| pH (0.01 CaCl_2_) | 0.17 | 0.20 | 0.35 | 0.23 |
| H (cmol_c_ kg^-1^) | 2.6 | 2.9 | 4.1 | 0.99 |
| Al sat. (%) | 8.1 | 1.5 | 24 | 6.9 |
| Al:C (g:g) | 0.0004 | 0.00007 | 0.0014 | 0.0003 |
| N (g kg^-1^) | 1.7 | 1.9 | 4.1 | 3.2 |
| N (Mg ha^-1^) | 0.22 | 0.33 | 0.45 | 0.30 |
| C:N (g:g) | 1.6 | 1.7 | 1.4 | 1.1 |
|  |  |  |  |  |
| **Oa Horizon** | **1997** | **2009-10** | **1998** | **2014** |
| C (g kg^-1^) | 85 | 72 | 88 | 76 |
| C Mg (ha^-1^) | 45 | 58 | 22 | 6.8 |
| CEC (cmol_c_ kg^-1^) | 6.1 | 6.5 | 7.4 | 3.6 |
| Base sat. (%) | 13 | 14 | 22 | 15 |
| pH (0.01 CaCl_2_) | 0.17 | 0.17 | 0.43 | 0.30 |
| H (cmol_c_ kg^-1^) | 4.5 | 3.9 | 3.8 | 2.0 |
| Al sat. (%) | 18 | 16 | 21 | 19 |
| Al:C (g:g) | 0.014 | 0.0011 | 0.0013 | 0.0007 |
| N (g kg^-1^) | 3.6 | 2.9 | 3.3 | 4.5 |
| N (Mg ha^-1^) | 1.3 | 1.8 | 0.91 | 0.36 |
| C:N (g:g) | 4.0 | 4.4 | 2.5 | 2.2 |
|  |  |  |  |  |
| **Upper B Horizon** | **1997** | **2009-10** | **1998** | **2014** |
| C (g kg^-1)^) | 33 | 28 | 24 | 24 |
| C (Mg ha^-1^) | 6.9 | 4.3 | 5.9 | 5.4 |
| CEC (cmol_c_ kg^-1^) | 1.6 | 3.4 | 0.94 | 2.0 |
| Base sat. (%) | 4.4 | 2.6 | 7.0 | 6.8 |
| pH (0.01 CaCl_2_) | 0.27 | 0.29 | 0.34 | 0.26 |
| H (cmol_c_ kg^-1^) | 0.9 | 1.1 | 0.58 | 0.42 |
| Al sat. (%) | 13 | 9.4 | 14 | 12 |
| Al:C (g:g) | 0.0026 | 0.0020 | 0.0011 | 0.0019 |
| N (g kg^-1^) | 1.6 | 1.5 | 0.99 | 1.2 |
| N (Mg ha^-1^) | 0.59 | 0.22 | 0.25 | 0.25 |
| C:N (g:g) | 10 | 4.6 | 4.8 | 3.1 |

**Table S2.** Standard deviation values for soil measurements determined in the initial and final samplings of North and South Buck watersheds for Oe, Oa, and upper 10 cm of the B horizon.

|  | **North Buck** | | **South Buck** | |
| --- | --- | --- | --- | --- |
| **Oe Horizon** | **1997** | **2009-10** | **1998** | **2014** |
|  | **CV** | **CV** | **CV** | **CV** |
| C (g kg^-1^) | 5 | 3 | 15 | 13 |
| C (Mg ha^-1^) | 18 | 24 | 38 | 34 |
| CEC (cmol_c_ kg^-1^) | 16 | 16 | 39 | 22 |
| Base sat. (%) | 20 | 19 | 44 | 15 |
| pH (0.01 CaCl_2_) | 6 | 6 | 11 | 7 |
| H (cmol_c_ kg^-1^) | 32 | 29 | 77 | 40 |
| Al sat. (%) | 88 | 58 | 93 | 202 |
| Al:C (g:g) | 79 | 54 | 103 | 208 |
| N (g kg^-1^) | 8 | 8 | 38 | 34 |
| N (Mg ha^-1^) | 16 | 22 | 15 | 14 |
| C:N (g:g) | 8 | 8 | 7 | 6 |
|  |  |  |  |  |
| **Oa Horizon** | **1997** | **2009-10** | **1998** | **2014** |
| C (g kg^-1^) | 21 | 15 | 22 | 21 |
| C Mg (ha^-1^) | 55 | 63 | 97 | 48 |
| CEC (cmol_c_ kg^-1^) | 20 | 21 | 34 | 23 |
| Base sat. (%) | 44 | 45 | 45 | 31 |
| pH (0.01 CaCl_2_) | 7 | 6 | 15 | 10 |
| H (cmol_c_ kg^-1^) | 42 | 30 | 81 | 44 |
| Al sat. (%) | 53 | 64 | 66 | 90 |
| Al:C (g:g) | 61 | 71 | 80 | 87 |
| N (g kg^-1^) | 21 | 15 | 16 | 24 |
| N (Mg ha^-1^) | 40 | 48 | 85 | 50 |
| C:N (g:g) | 16 | 19 | 13 | 11 |
|  |  |  |  |  |
| **Upper B Horizon** | **1997** | **2009-10** | **1998** | **2014** |
| C (g kg^-1)^) | 41 | 30 | 43 | 41 |
| C (Mg ha^-1^) | 19 | 35 | 22 | 28 |
| CEC (cmol_c_ kg^-1^) | 32 | 32 | 29 | 45 |
| Base sat. (%) | 38 | 44 | 48 | 64 |
| pH (0.01 CaCl_2_) | 8 | 8 | 9 | 7 |
| H (cmol_c_ kg^-1^) | 67 | 74 | 111 | 110 |
| Al sat. (%) | 21 | 12 | 19 | 14 |
| Al:C (g:g) | 60 | 23 | 29 | 32 |
| N (g kg^-1^) | 45 | 42 | 38 | 45 |
| N (Mg ha^-1^) | 12 | 17 | 20 | 29 |
| C:N (g:g) | 41 | 17 | 23 | 13 |

**Table S3.** Values of CV (coefficient of variation) for soil measurements of the initial and final samplings of North and South Buck watersheds for Oe, Oa, and upper 10 cm of the B horizon.
